# Mechanistic study on electroacupuncture-regulated circadian autophagy for inhibiting ferroptosis in hippocampal neurons and alleviating depression-like behaviors in adulthood induced by early chronic sleep deprivation

**DOI:** 10.3389/fneur.2025.1680606

**Published:** 2025-11-07

**Authors:** Yuzhu Wu, Xinwang Chen, Feixiang Liu, Yanchen Feng, Ziyun Liao, Xinyu Bu, Wen Fu, Jing Gao, Lihua Wu, Linyuan Fan, Qian Yang, Penglin Yue, Mengyu Wang

**Affiliations:** 1 School of Rehabilitation Medicine, Henan University of Chinese Medicine, Zhengzhou, China; 2 College of Acupuncture, Moxibustion and Tuina, Henan University of Chinese Medicine, Zhengzhou, China; 3 Department of Neurology, Psychiatry and Psychology, The First Affiliated Hospital of Henan University of Chinese Medicine, Zhengzhou, China; 4 Henan Collaborative Innovation Center for the Prevention and Treatment of Major Diseases with Traditional Chinese and Western Medicine, Zhengzhou, China; 5 The First Clinical Medical College of Henan University of Chinese Medicine, Zhengzhou, China; 6 Zhengzhou People's Hospital, Zhengzhou, China; 7 New Universe Acupuncture Inc., Pasadena, CA, United States; 8 The First Affiliated Hospital of Henan University of Chinese Medicine, Zhengzhou, China; 9 The Second Clinical Medical College of Henan University of Chinese Medicine, Zhengzhou, China

**Keywords:** chronic sleep deprivation, depression, electroacupuncture, ferroptosis, hippocampal neurons, clock-controlled autophagy

## Abstract

Early-life chronic sleep deprivation (CSD) represents a significant risk factor for the development of adult depressive-like behaviors, although the precise molecular mechanisms underlying this association remain incompletely elucidated. In this study, we established a CSD rat model using a rodent sleep deprivation apparatus and employed behavioral tests to assess the effects of early electroacupuncture intervention on adult depressive-like behaviors. The underlying molecular mechanisms were systematically investigated through integrated experimental approaches including Prussian blue staining, transmission electron microscopy, enzyme-linked immunosorbent assay, and Western blot analysis. The experimental results demonstrated that the CSD group rats exhibited significant depressive-like behaviors, accompanied by pathological alterations such as increased iron deposition in hippocampal tissues and structural damage to neuronal mitochondria. Electroacupuncture intervention facilitated hippocampal neuronal repair and remodeling through multiple mechanisms, including the regulation of iron metabolism homeostasis, enhancement of antioxidant capacity, inhibition of ferroptosis, and suppression of excessive autophagy, thereby reversing the CSD-induced depressive-like behaviors. This study provides novel therapeutic strategies and mechanistic insights for the prevention and treatment of adult-onset depression induced by early-life chronic sleep deprivation.

## 1 Introduction

Chronic sleep deprivation (CSD), a widespread and disconcerting issue in today's world, is marked by diminished total sleep duration or by frequent, fleeting awakenings that disrupt a solid night's rest. As a result, people are left with insufficient sleep that doesn't allow for proper rejuvenation, leading to decreased alertness and compromised behavioral abilities (1). Research has revealed that CSD not only boosts the likelihood of developing depression but also intensifies the symptoms for those who are already grappling with the condition (2). Adolescence is a critical period of brain development and cognitive function formation, during which sleep is vital. CSD during adolescence can exert adverse and long-lasting effects on the central nervous system. Our previous research demonstrated that early CSD resulted in depression in adulthood. Animal studies similarly showed that CSD during adolescence caused learning and memory function impairments and depression-like behaviors in adulthood (3). Studies have shown that individuals with insufficient sleep have ~60% increased activation of the amygdala—the brain's emotional center—when exposed to negative emotional stimuli, indicating that CSD significantly impairs emotional regulation (4). Therefore, exploring safer and more effective therapies to treat depression remains an urgent challenge.

The circadian clock-regulated autophagy-dependent (CC-ATG) ferroptosis pathway represents an iron-mediated mechanism of programmed cellular demise. Its hallmark features include the depletion of glutathione (GSH) and diminished activity of glutathione peroxidase 4 (GPX4). As this protective system falters, GPX4 loses its ability to neutralize lipid peroxides through GSH reduction. Consequently, iron-dependent lipid oxidation escalates, producing harmful reactive oxygen species (ROS). These unstable molecules inflict oxidative stress that culminates in irreversible cell death (5). Studies have found that autophagy is a critical regulator of ferroptosis. Autophagy modulates ferroptosis through multiple pathways, including ferritinophagy, which selectively degrades ferritin, releases Fe^2+^ to promote lipid peroxidation, and directly triggers ferroptotic cell death (6). Autophagy plays an indirect role in regulating ferroptosis by tweaking GPX4 levels, which in turn affects how well mitochondria function and their ability to mop up reactive oxygen species. What's more, your body's internal clock, the circadian rhythm, keeps iron levels in check by managing the expression of key autophagy genes (like *Beclin1, ATG5*, and *LC3*) (7). Circadian genes such as *ARNTL* and *CLOCK* upregulate and downregulate the transcriptional levels of autophagy-related proteins, thereby mediating the cell's response to oxidative stress and iron load (8). Therefore, disrupting the circadian rhythm interferes with autophagy regulation, which in turn leads to iron metabolism abnormalities and ultimately ferroptosis. Research indicates that CC-ATG ferroptosis plays a significant role in the onset of depression, disrupting hippocampal and prefrontal cortex activity by triggering neuroinflammatory responses (9). MRI scans with quantitative susceptibility mapping revealed a notable increase in iron buildup within areas like the hippocampus and prefrontal cortex in those suffering from chronic depression. This finding hints that the buildup of iron could be a telltale sign of brain damage in cases of recurring depression, as per the research (10). Therefore, abnormal iron metabolism in the neurons of depression patients leads to increased oxidative stress, catalyzing the Fenton reaction and generating hydroxyl radicals, which ultimately cause intracellular oxidative damage (11). This was shown to underlie behaviors associated with anhedonia in depression patients (12). In summary, the circadian rhythm, autophagy, and ferroptosis form an important regulatory axis that participates in the onset and progression of depression. Gaining more profound insight into how this regulatory system functions could shed light on the underlying pathology of adult-onset depression triggered by early-life circadian rhythm disruption. Such knowledge would pave the way for innovative therapeutic approaches, including restoring biological rhythms, regulating autophagy processes, and blocking ferroptosis pathways.

In recent years, electroacupuncture has demonstrated significant clinical effects as a treatment for depression. According to traditional Chinese medicine theory, electroacupuncture regulates the flow of “liver qi” and balances “yin–yang,” which may contribute to the alleviation of depressive symptoms. A clinical study employing randomized controlled trial methodology examined whether electroacupuncture could effectively alleviate insomnia linked to depression. The findings revealed that patients receiving electroacupuncture demonstrated marked improvements in sleep quality compared to the control group. Both the Pittsburgh Sleep Quality Index and Hamilton Depression Rating Scale scores showed statistically significant enhancements, indicating that electroacupuncture may offer a viable treatment option for sleep disturbances associated with depressive disorders (13). Electroacupuncture significantly improves sleep quality in depression patients and also effectively alleviates anxiety and depressive symptoms. In a study involving individuals addicted to methamphetamine, electroacupuncture notably improved the mental state of patients and reduced anxiety and depression scores, demonstrating the broad applicability of electroacupuncture in various psychiatric disorders (14). Electroacupuncture has also been shown to significantly inhibit ferroptosis by activating the autophagy pathway. Studies found that electroacupuncture significantly increased levels of autophagy markers such as Beclin1 in the hippocampus of type 2 diabetic mice, while reducing levels of p-mTOR, indicating that electroacupuncture activated autophagy by inhibiting mTOR. Additionally, the autophagy inhibitor 3-MA was shown to reverse the protective effects of electroacupuncture on neuronal ferroptosis and cognitive function (15). Research has demonstrated that the use of electroacupuncture can thwart ferroptosis in hippocampal neurons, doing so by curbing the buildup of iron and preventing lipid oxidation, while also bolstering the body's antioxidant defenses (16). This, in turn, has been linked to an improvement in depressive symptoms among individuals suffering from post-stroke depression (17). In addition, acupuncture prevented cellular ferroptosis and improved depression-like behaviors in rats by increasing serum GSH levels and reducing the activation of hippocampal microglial cells. However, it remains unclear whether electroacupuncture can improve depression caused by early CSD in adulthood by inhibiting ferroptosis in hippocampal neurons and promoting hippocampal neuronal synaptic remodeling. Therefore, this study investigated the mechanism by which electroacupuncture alleviated depression induced by childhood-to-adolescent CSD in adulthood, with a focus on ferroptosis regulation in hippocampal neurons. By diving into alterations in iron metabolism, antioxidant defenses, indicators of lipid peroxidation, and the expression of proteins tied to ferroptosis within the hippocampus, this study sought to shed light on precisely how electroacupuncture alleviates depression stemming from early-life CSD in adulthood. This investigation lays a theoretical groundwork for using electroacupuncture to manage depression triggered by early CSD, while also offering a fresh angle on the involvement of ferroptosis in this particular condition.

## 2 Materials and methods

### 2.1 Study subjects

**Figure 1:**
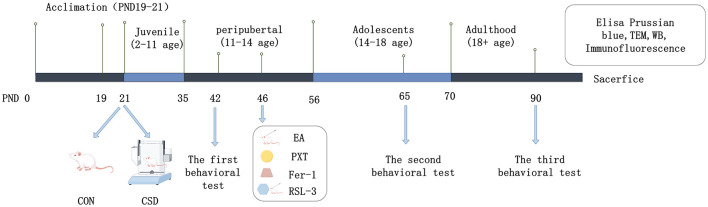
Experimental procedure diagram.

For this study, we used 72 young male Wistar rats, all of specific pathogen-free (SPF) grade and weighing between 40 and 80 grams. We sourced these critters from Beijing Vital River Laboratory Animal Technology Co., Ltd. [Production License No.: SYXK (Beijing) 2021-0006]. All animal-related procedures strictly adhered to the ethical guidelines outlined in China's Ministry of Science and Technology 2006 Laboratory Animal Care and Use Policy. The subjects were housed in Henan University of Chinese Medicine's state-of-the-art SPF-certified animal facility, where environmental conditions were meticulously controlled—maintaining temperatures between 20 and 22°C, humidity levels at 40–60%, and a regulated 12-h light/dark circadian cycle. The CSD cohort was subjected to uninterrupted light exposure throughout the sleep deprivation phase. Animals had free access to food and water, with bedding refreshed every 72 h to maintain hygiene. To avoid solitary stress, a minimum of two rats shared each enclosure. Following a seven-day acclimatization period with standardized feeding, subjects were randomly assigned via computerized randomization to either the control (CON) group (12 animals) or the CSD group (60 animals). Figure 1 provides a schematic overview of the experimental protocol.

### 2.2 Model establishment and grouping

CSD modeling in the CSD group was performed using a small animal sleep deprivation apparatus (Model ZL013, Anhui Yaokun Biotechnology Co., Ltd.). From postnatal day (PND) 19–21, rats underwent 1-h daily adaptation to sleep restriction for three consecutive days within the testing apparatus. From PND 21–42, CSD was formally induced in the rats using the sleep deprivation apparatus. The main chamber of the device was equipped with food and water dispensers on the side, allowing the rats to eat and drink freely. Sleep deprivation was scheduled from 00:00 to 10:00 and from 16:00 to 24:00 each day, for a total of 18 h of deprivation per day. The rotational speed of the apparatus was set to 5.0 rpm. For the subsequent 6 h daily (10:00–16:00), rodents were placed back in enclosures for unrestricted rest. Rats were exposed to formal sleep deprivation for a total of 21 days. During this period, rats in the CON group were maintained under standard housing conditions. The lab's temperature was kept steady between 20 and 22 degrees Celsius, and the lights were always on for the entire experiment. Once the model was set up, the 60 rats suffering from CSD were split up randomly into five different groups (a dozen in each): the CSD group, a group getting electroacupuncture (EA), a group on paroxetine (PXT), a positive control group using ferrostatin-1 (Fer-1), and lastly, a group where ferroptosis was triggered using RSL-3, combined with electroacupuncture (RSL-3). Our animal housing and testing protocols strictly adhered to the established guidelines laid out in the GB/T 35892-2018 Standards for Ethical Review of Laboratory Animal Welfare. The research was meticulously reviewed and received approval from Henan University of Chinese Medicine's Institutional Animal Care and Use Committee, with the following official number: IACUC-202310022.

Seventy-two male Wistar rats were randomly divided into six groups: CON, CSD, EA, PXT, Fer-1, and RSL-3 (*n* = 12 per group). Sample size was determined using G^*^Power 3.1 based on preliminary data and previous studies, with sucrose preference as the primary indicator (effect size *d* = 1.2, α = 0.05, power = 0.8). The calculated minimum sample size was 10 per group, and 12 were included to compensate for potential loss. All behavioral tests were conducted on the full sample, while three rats per group were randomly selected for molecular and histological analyses (Western blot, ELISA, iron staining, TEM, and confocal imaging), following the 3R principle.

### 2.3 Main reagents and instruments

Paroxetine hydrochloride tablets were purchased from Wan Sheng Li Le (Beijing Wan sheng Pharmaceutical Co., Ltd., Beijing, China). We procured Tribromoethanol (T903147) from Shanghai Macklin Biochemical Co., Ltd., located in Shanghai, China. For Ferrostatin-1 (S8155), we turned to Selleck Chemicals, based out of Houston, Texas, in the United States. Lastly, we sourced RSL-3 (F129882) from Aladdin, a supplier situated in Shanghai, China. Paraformaldehyde universal tissue fixative (4%), a BCA protein concentration assay kit, and a Prussian blue staining kit were purchased from Solarbio (Beijing, China). A ferrous ion (Fe^2+^) colorimetric assay kit, glutathione ELISA kit, and malondialdehyde (MDA) ELISA kit were purchased from Elabscience (Wuhan, China). A PAGE gel preparation kit and tri-color pre-stained protein marker were purchased from Yeasen (Shanghai, China). The following primary and secondary antibodies were employed in the study: ARNTL (14268-1-AP, Proteintech, Wuhan), CLOCK (GB111884, Solarbio, Beijing), FTH1 (11682-1-AP, Proteintech), FTL (10991-1-AP, Proteintech), GPX4 (GB154327, Solarbio), LPCAT3 (67882-1-Ig, Proteintech), ACSL4 (81196-1-RR, Proteintech), LOX (17958-1-AP, Proteintech), SLC7A11 (GB115276, Solarbio), and β-actin (14268-1-AP, Proteintech) as the loading control. For detection, HRP-conjugated secondary antibodies were used: goat anti-rabbit (GB23303, Solarbio) and goat anti-mouse (GB23301, Solarbio). All antibody manufacturers were based in China, with Proteintech located in Wuhan and Solarbio in Beijing.

Disposable sterile acupuncture needles and an SDZ-II electronic acupuncture stimulator were purchased from Huatuo (Suzhou, China). A SuperFst high-throughput forced swim test system was purchased from XinRuan (Shanghai, China). An automatic sucrose preference detection system was provided by LogiScience (Singapore). An OFT-100 open field test system for rats and mice was provided by Taimeng (Chengdu, China). Our research also relied on a variety of instruments. These included a high-speed centrifuge from Eppendorf (Hamburg, Germany), and for sectioning, we used a Dakewei semi-automatic rotary microtome (Shenzhen, China). A Thermo Fisher Scientific microplate reader (Waltham, MA, USA) was essential for our assays, as was a Bio-Rad mini vertical electrophoresis and transfer system (Hercules, CA, USA) for protein analysis. We visualized our results using a Vilber chemiluminescence imaging system (France). Tissue processing involved a Dakewei tissue dehydrator and rotary microtome (Shenzhen, China) alongside a Precision Star paraffin embedding machine (Changzhou, China). Finally, microscopy was performed using both a Nikon optical microscope (Tokyo, Japan) and a Hitachi transmission electron microscope (Tokyo, Japan) for detailed structural analysis.

### 2.4 Intervention methods

In the EA group, four acupoints were selected for the study: Baihui (GV20), Yintang (GV29), Hegu (LI4), and Taichong (LR3). The acupoint selection was based on the 14th *Five-Year Plan Textbook of Acupuncture and Moxibustion* edited by Liang Fanrong and Zhao Jiping. Acupoint localization was performed with reference to “Nomenclature and Location of Commonly Used Acupoints in Laboratory Animals, Part II: Rat.” GV20, known as Baihui, is located along the midline of the rat's parietal bone; GV29, or Yintang, rests precisely at the midpoint where the peaks of both orbital rims meet; Hegu, at LI4, can be found sandwiched between the first and second bones of the forelimb's metacarpals; and LR3, or Taichong, lies nestled in the hollow space between the first and second bones on the top of the hindlimb. In a controlled setting, the rodents were secured on a specialized rat grip, lying belly-down, and the skin areas for acupuncture were meticulously sanitized. During the procedure, pre-sterilized, single-use, stainless steel needles measuring 0.20 mm in diameter and 13 mm in length were applied. The needles were placed at Hegu and Taichong points straight into the skin, to a depth of 2 mm, and this was done on both sides alternately. For the Baihui and Yintang points, the needles were inserted at a 15-degree angle to the skin, reaching a depth of 2 mm—no electrical stimulation was administered, and the needles were left in place. The electroacupuncture device (SDZ-II, manufactured by Suzhou Huatuo in Suzhou, China) was set up with its anode and cathode attached to the Hegu and Taichong points on the same side of the body. Using intermittent pulses at frequencies ranging from 2 to 10 Hz and currents between 1 and 1.2 mA, the stimulation intensity was carefully calibrated until the rat showed no signs of distress or resistance. Each session lasted 20 min and was conducted once daily for 18 consecutive days, from PND 46 to PND 64. Following the completion of the modeling phase, rats in the PXT group received daily oral doses of PXT (10 mg/kg) over an 18-day period. Similarly, the Fer-1 group was administered Fer-1 (20 mg/kg) via intraperitoneal injection once per day for the same duration. In the RSL-3 group, rats were given intraperitoneal injections of RSL-3 (100 mg/kg) followed by daily electroacupuncture sessions, mirroring the protocol used in the EA group. Meanwhile, the CON, CSD, PXT, and Fer-1 groups underwent identical handling and restraint procedures as the electroacupuncture groups each day—just without the electrical stimulation component—to ensure consistent experimental conditions.

### 2.5 Behavioral testing of rats

#### 2.5.1 Sucrose preference test (SPT)

Testing was performed on PND 42, PND 65, and PND 90. Prior to the test, rats were housed individually and underwent a 48-h sucrose drink training period. In the initial 24-h period, each rat had access to two bottles containing a 2% sucrose solution. Subsequently, over the following 24 h, they were presented with one bottle of the 2% sucrose solution alongside a bottle of plain water. To nix any positional bias, the bottles were switched around every 12 h. After the acclimation phase, the volume of sucrose and water that each rat drank was monitored for 12 h. Because rats appeared more active and consumed more food and water during the night, the sucrose preference was measured during their active period (20:00–8:00) for more accurate results. Sucrose Preference Ratio = (Sucrose Consumption/Total Fluid Consumption) × 100%.

#### 2.5.2 Open-field test (OFT)

We ran the OFT on PND 43, PND 66, and PND 91. Before each session, rats got a 30-min acclimation period to get used to the testing environment. When it was go-time, we placed each rat in the center of the open field. For the next 5 min, we tracked things like the total distance they covered, how many times they crossed grid lines, how far they ventured into the center, and how long they hung out there. To maintain the integrity of the experiment and ensure reproducibility, the equipment was thoroughly wiped with 75% ethanol after each session to remove any odor traces that could affect subsequent tests.

#### 2.5.3 Novelty-suppressed feeding test (NSFT)

Testing was conducted on PND 44, PND 67, and PND 92. Prior to the experiment, the rats were food-deprived for 24 h. During the experiment, a small amount of food was placed in the center of an 80 × 80 × 40 cm open-field box. The rodent was subsequently positioned in the box's stationary corner. The time from when the rat entered the field to when it picked up the food and began eating was recorded as the latency to feed. For rats that did not begin eating within 6 min, the latency was recorded as 6 min. Afterward, the rat was returned to its cage, and the amount of food consumed during the following 5 min was recorded to eliminate the potential effects of appetite differences on the results.

#### 2.5.4 Forced swim test (FST)

Testing was conducted on PND 45, PND 68, and PND 93. On the day prior to testing, the rats underwent a 15-min acclimation swim in a clear cylindrical tank. Measuring 30 cm across and 60 cm tall, the container held water at a depth of 33 cm—just deep enough to prevent the animals from touching the bottom with their hind paws. The water was kept at a consistent 23°C, give or take 2 degrees. Exactly one day later, the actual forced swim test took place in identical conditions. During this 6-min trial, which was recorded on video, researchers analyzed the final 4 min of inactivity. An independent observer, unaware of which experimental group each rat belonged to, evaluated the immobility periods. Floating or only slight movements to maintain balance were considered as immobility. The procedure was meticulously documented and evaluated using a SMART 3.0 video tracking system (Reward). Over the final 4 min of the trial, the duration of immobility, active swimming, and frantic struggling was measured to assess the extent of helplessness exhibited. To eliminate any potential carryover effects, the water in the tank was refreshed after each rat underwent the test, ensuring unbiased conditions for the next subject.

### 2.6 Prussian blue staining for observing hippocampal tissue iron deposition

The paraffin-embedded tissue samples underwent deparaffinization and rehydration before being thoroughly coated with Prussian blue stain. These prepared sections were then placed in a humidified chamber at 37°C for a 20-min incubation period, followed by three distilled water rinses. Subsequently, an incubation solution was introduced, and the samples were returned to the humidified environment at 37°C for another 15 min. After triple washing with Phosphate-Buffered Saline, an enhancer solution was carefully applied before a final 20-min incubation at 37°C. The process concluded with a 10-min distilled water wash, followed by dehydration, clearing, and mounting of the tissue sections. Iron deposition in the hippocampal tissue was observed under an optical microscope.

### 2.7 Transmission Electron Microscopy (TEM) for observing the mitochondrial ultrastructure of hippocampal neurons

The hippocampal tissue was swiftly frozen on ice, then meticulously chopped into 1 mm^3^ cubes and preserved in a 2.5% glutaraldehyde solution. Next, it was dehydrated through a series of acetone concentrations before being embedded in epoxy resin. The tissue was then thinly sliced to just 50 nm. These slices were stained with uranyl acetate and lead citrate, allowing for a detailed examination of the hippocampal neurons' mitochondrial ultrastructure via a transmission electron microscope.

### 2.8 Fe^2+^ colorimetric assay for measuring Fe^2+^ content of hippocampal tissue

Hippocampal tissue was collected from the rats, and the tissue samples were homogenized using a ratio of 1 g tissue to 9 mL reagent at a low temperature. Following a brisk spin in a refrigerated centrifuge (4°C at 12,000 × g for 10 min), we collected the supernatant. A microplate reader then gave us the absorbance readings at 593 nm, which we used to figure out the tissue's Fe^2+^ levels.

### 2.9 ELISA for measuring GSH and MDA levels in hippocampal tissue

Hippocampal tissue from each group of rats was collected and homogenized using a ratio of 1 g tissue to 9 mL PBS at a low temperature. Following a spin in the centrifuge at a brisk 5,000 × g for 10 min at a chilly 4°C, the liquid on top was carefully siphoned off. We then proceeded to measure GSH and MDA levels in the hippocampus, sticking closely to the protocol detailed in the ELISA kit's instructions. A microplate reader was used to get absorbance readings at 450 nm, and these values were then plugged into standard curves to figure out the GSH and MDA concentrations in the hippocampal tissue.

### 2.10 Western blot for measuring ferroptosis, circadian clock, and lipid oxidative stress-related protein levels in hippocampal tissue

Rat hippocampal tissue samples were harvested, and total protein was isolated for analysis. Protein concentrations were quantified via the BCA assay. For electrophoresis, 5 μL of each sample was loaded and run at 200 V for half an hour, after which proteins were transferred to a membrane at 300 mA for another 30 min. The membrane was rinsed briefly with Tris-Buffered Saline containing Tween-20 (TBST) and then blocked for 2 h using 5% non-fat dry milk. Following an 8-min TBST wash, the membrane was incubated at 4°C overnight with primary antibodies—ARNTL, CLOCK, FTH1, FTL, SLC7A11, LPCAT3, GPX4, ACSL4, and LOX—all diluted at 1:2000. The membrane underwent a thorough cleaning process, getting rinsed three times with TBST for a solid 8 min each round. Next, the appropriate HRP-linked secondary antibodies were introduced—the mix included goat anti-rabbit at a 1:5000 dilution and rabbit anti-mouse at the same concentration. The membrane was then allowed to incubate at a toasty 37°C for a full hour. Post-washing, once more with TBST, the membrane was treated with an enhanced chemiluminescence reagent. The resultant protein bands were captured via a nifty chemiluminescence imaging rig. Band intensity was meticulously examined with the help of ImageJ software. The target protein's relative expression was computed by dividing the target band's intensity by that of the internal control. The data was also normalized against the CON group.

### 2.11 Confocal laser scanning microscopy for observing co-localization of the circadian clock autophagy protein ARNTL and autophagosomes

In our histological analysis, we began by deparaffinizing and rehydrating the 3-μm paraffin sections and then subjected them to antigen retrieval. Once the sections were left to air dry for a brief moment, we meticulously outlined the tissue area with a hydrophobic pen. Next, we applied a 5% BSA blocking solution to the sections, allowing them to incubate at a cozy 37°C for a solid hour. Once the blocking process was complete, we introduced the primary antibody (BMAL1, diluted 1:200) and kept the sections chilling in the fridge overnight at 4°C. Upon the return of day, we brought the sections up to room temperature, treated them with the matching secondary antibody (HRP-conjugated goat anti-rabbit, also at 1:200), and applied the dye. Post-antibody removal, we added a second primary antibody (LC3B, at 1:500) and then followed up with the appropriate secondary antibody. The sections were then treated with dye and left to incubate at room temperature, shielded from light, for a 10-min period. Following this step, the nuclei were counterstained with DAPI for 8 min. Next, the samples underwent a series of washes with distilled water before being dehydrated, cleared, and finally mounted for analysis. Using an inverted fluorescence microscope, researchers examined the co-localization of ARNTL with autophagosomes and recorded the resulting images.

### 2.12 Statistical methods

Quantitative results are presented as the mean ± SEM. We crunched the numbers using GraphPad Prism (version 9.4.0) and SPSS (version 25.0) for all statistical analyses. To check if our data were normally distributed, we performed a Shapiro-Wilk test. For data that played by the rules and followed a normal distribution, we performed a one-way ANOVA, followed by Tukey's *post-hoc* test to tease out any differences between groups. If the data went rogue and didn't conform to a normal distribution, we turned to the Kruskal-Wallis H test, with Dunn's *post-hoc* analysis to pinpoint significant variations. We considered anything with a *P*-value < 0.05 to be statistically significant.

## 3 Results

### 3.1 Behavioral assessment of depression-like symptoms in rats

#### 3.1.1 Childhood to adolescence (PND 21 to PND 42) CSD rat model construction

**Figure 2:**
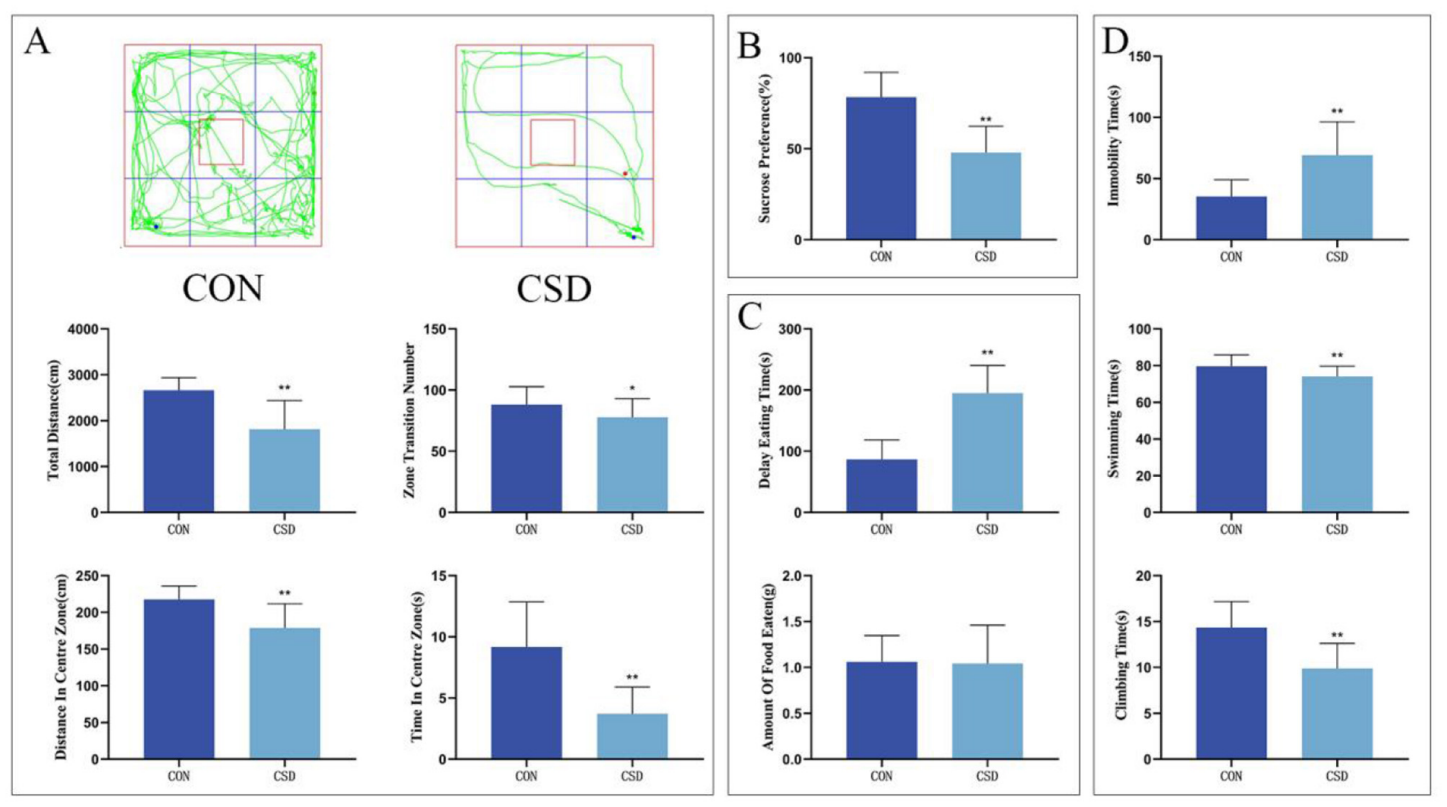
**(A)** From top to bottom: OFT trajectory maps of rats in each group on PND 43, total distance traveled by rats in the open field, total number of grid crossings by rats in the open field, total distance traveled by rats in the central area, and time spent by rats in the central area. **(B)** Sucrose preference rates of rats in each group on PND 42. **(C)** Latency to feed and amount of food consumed by rats on PND 44. **(D)** Immobility ratio, swimming duration ratio, and struggling duration ratio of rats on PND 45 (CON group, *n* = 12; CSD group, *n* = 60). *P < 0.05, **P < 0.01 compared with the CON group.

SPT findings revealed a notably decreased sucrose preference in the CSD cohort vs. the CON cohort on PND 42 (*P* < 0.01) (as shown in Figure 2B).

The OFT results related to PND 43 indicated that the CSD group showed a marked decrease compared to the CON group in several key measures: they did not travel as far overall, didn't cross grid lines as often, kept to the edges more, and didn't venture into the center as much (*P* < 0.01, *P* < 0.05) (as shown in Figure 2A).

NSFT outcomes at PND 44 revealed markedly delayed feeding initiation in the CSD cohort vs. the CON cohort (*P* < 0.01) (as shown in Figure 2C).

On PND 45, the FST results revealed striking behavioral differences between the groups. Mice in the CSD cohort spent markedly more time immobile (*P* < 0.01) while showing significantly less swimming and struggling activity (*P* < 0.01) compared to the CON group. These findings clearly demonstrate the profound impact of CSD on depressive-like behaviors (as shown in Figure 2D).

The remaining comparisons showed no significant differences.

#### 3.1.2 Adolescence to adulthood (PND 46 to PND 64) intervention in CSD rats

**Figure 3:**
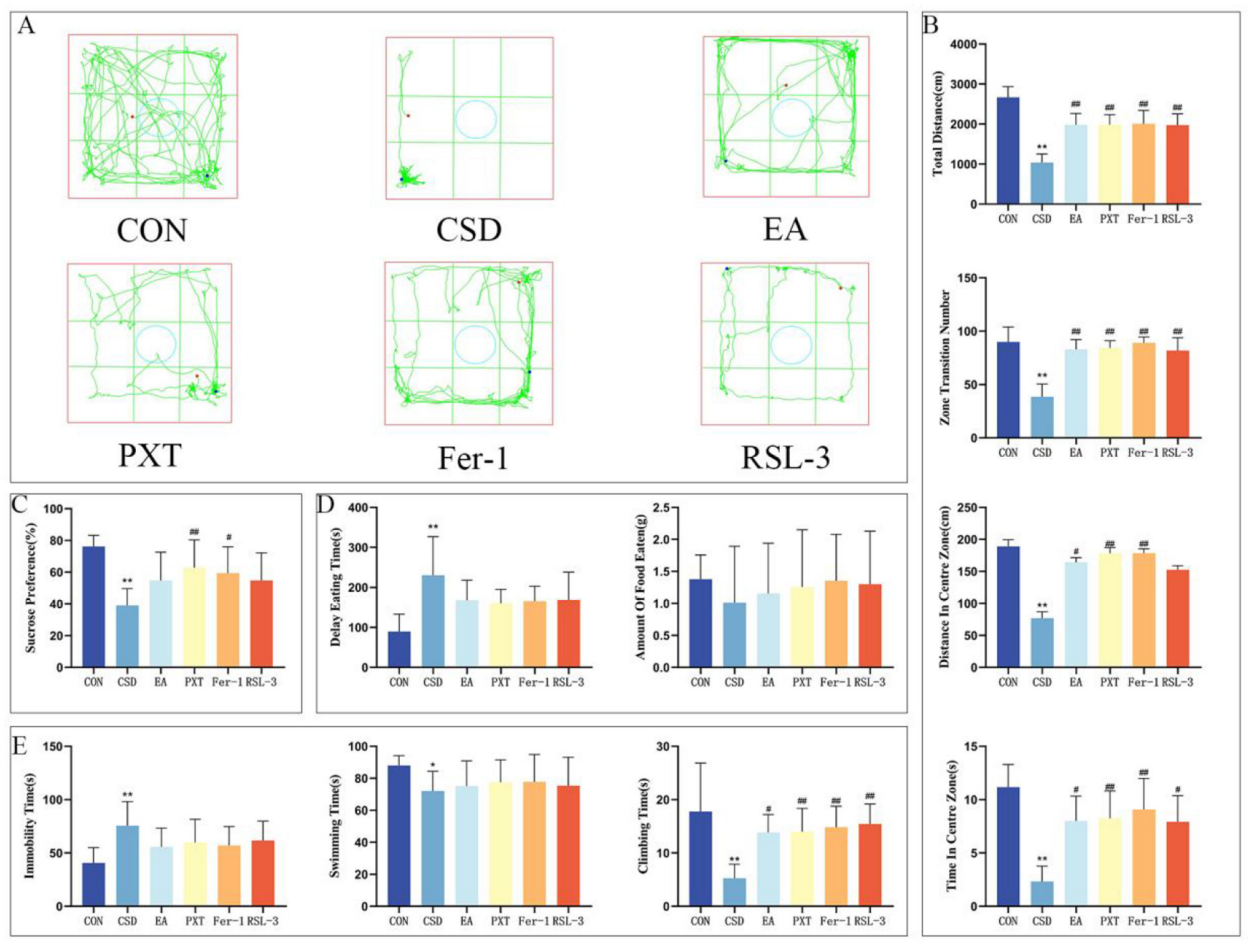
**(A)** OFT trajectory maps of rats in each group on PND 66. **(B)** Total distance traveled by rats in the open field, total number of grid crossings by rats in the open field, total distance traveled by rats in the central area, and time spent by rats in the central area on PND 66. **(C)** Sucrose preference rates of rats in each group on PND 65. **(D)** Latency to feed and amount of food consumed by rats on PND 67. **(E)** Immobility ratio, swimming duration ratio, and struggling duration ratio of rats on PND 68. *n* = 12 per group. *P < 0.05, **P < 0.01 compared with the CON group; ^#^*P* < 0.05, ^##^*P* < 0.01 compared with the CSD group.

The SPT results on PND 65 revealed a significantly lower sucrose preference in the CSD group vs. CON (*P* < 0.01). The PXT and Fer-1 groups exhibited a notably greater sucrose preference than the CSD group (*P* < 0.01 and *P* < 0.05, respectively)(as shown in Figure 3C).

The findings from the OFT conducted on PND 66 revealed a considerable reduction in the total distance covered, total grid crossings, the distance traversed in the central zone, and the duration spent in the central area for the CSD group as opposed to the CON group, with these differences being statistically significant (*P* < 0.01). The EA, PXT, Fer-1, and RSL-3 groups showed markedly higher activity levels than the CSD group, covering greater distances overall, crossing more grid lines, and spending more time and distance in the central zone (*P* < 0.01, *P* < 0.05). The differences were statistically significant, highlighting a clear contrast in movement patterns between the groups (as shown in Figures 3A, B).

On PND 67, NSFT findings revealed notably prolonged feeding latency in the CSD cohort vs. the CON cohort (*P* < 0.01) (as shown in Figure 3D).

The results of the FST on PND 68 showed that compared to the CON group, the CSD group had a significantly increased floating time (*P* < 0.01) and significantly decreased swimming and struggling times (*P* < 0.01, *P* < 0.05, respectively). EA, PXT, Fer-1, and RSL-3 groups showed markedly longer struggle durations compared to the CSD group (*P* < 0.01, *P* < 0.05) (as shown in Figure 3E).

Other contrasts showed no statistical importance.

#### 3.1.3 Late adulthood (PND 69 to PND 90)

**Figure 4:**
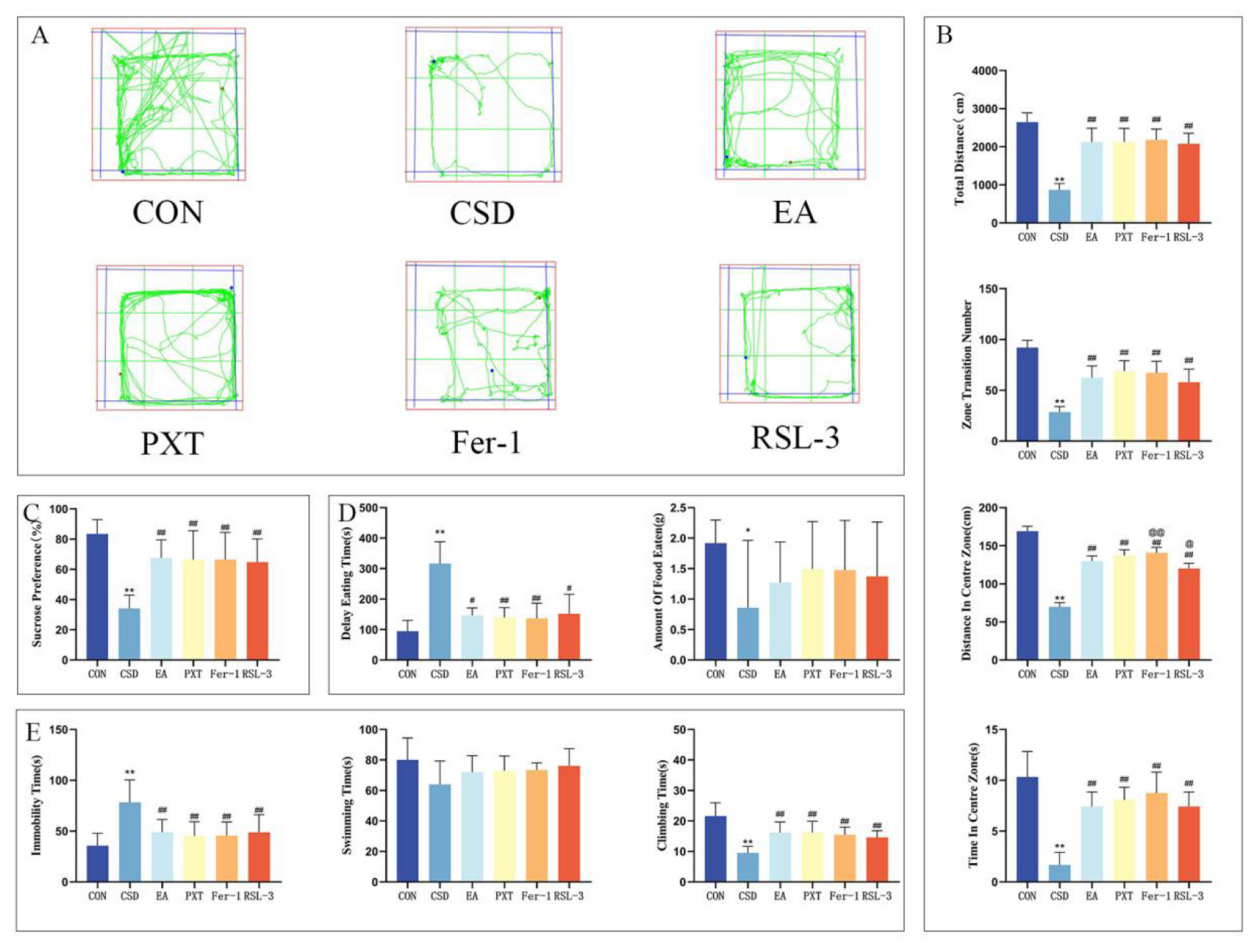
**(A)** OFT trajectory maps of rats in each group on PND 91. **(B)** Total distance traveled by rats in the open field, total number of grid crossings by rats in the open field, total distance traveled by rats in the central area, and time spent by rats in the central area on PND 91. **(C)** Sucrose preference rates of rats in each group on PND 90. **(D)** Latency to feed and amount of food consumed by rats on PND 92. **(E)** Immobility ratio, swimming duration ratio, and struggling duration ratio of rats on PND 93. *n* = 12 per group.*P < 0.05, **P < 0.01 compared with the CON group; ^#^*P* < 0.05, ^##^*P* < 0.01 compared with the CSD group; ^@^*P* < 0.05, ^@@^*P* < 0.01 compared with the EA group.

SPT findings at PND 90 indicated a notably decreased sucrose preference in the CSD group relative to the CON group (*P* < 0.01). The EA, PXT, Fer-1, and RSL-3 groups showed a significantly greater sucrose preference than the CSD group (*P* < 0.01) (as shown in Figure 4C).

The findings from the OFT on PND 91 revealed a notable reduction in the CSD group's total distance covered, total grid crossings, and the distance traveled within the central zone, as well as the time spent there, when contrasted with the CON group. This difference was statistically significant (*P* < 0.01). The EA, PXT, Fer-1, and RSL-3 groups showed markedly greater overall movement, with significantly higher total distances covered, more grid crossings, increased exploration of the central zone, and longer durations spent in the center compared to the CSD group (*P* < 0.01). The Fer-1 group exhibited a markedly greater distance covered in the central zone compared to the EA group (*P* < 0.01), whereas the RSL-3 group showed a statistically significant reduction in movement within the same area (*P* < 0.05) (as shown in Figures 4A, B).

When we ran the NSFT on PND 92, the CSD group took considerably longer to start eating (*P* < 0.01) and didn't eat nearly as much (*P* < 0.05) compared to the CON group. On the flip side, the EA, PXT, Fer-1, and RSL-3 groups showed a marked decrease in how long it took them to begin feeding compared to the CSD group (*P* < 0.01, *P* < 0.05) (as shown in Figure 4D).

On PND 93, the FST results showed notable group variations in behavior. The CSD group exhibited markedly longer periods of immobility (*P* < 0.01) and shorter active struggling phases (*P* < 0.01) when measured against the control animals. However, the EA, PXT, Fer-1, and RSL-3 treatment groups showed a complete reversal of this pattern—demonstrating significantly reduced floating durations (*P* < 0.01) and substantially increased escape-oriented behaviors (*P* < 0.01) compared to the CSD cohort. These findings suggest the therapeutic interventions effectively counteracted the depression-like symptoms observed in the CSD model (as shown in Figure 4E).

Other contrasts showed no statistical importance.

### 3.2 Comparison of hippocampal iron deposition in rats

**Figure 5:**
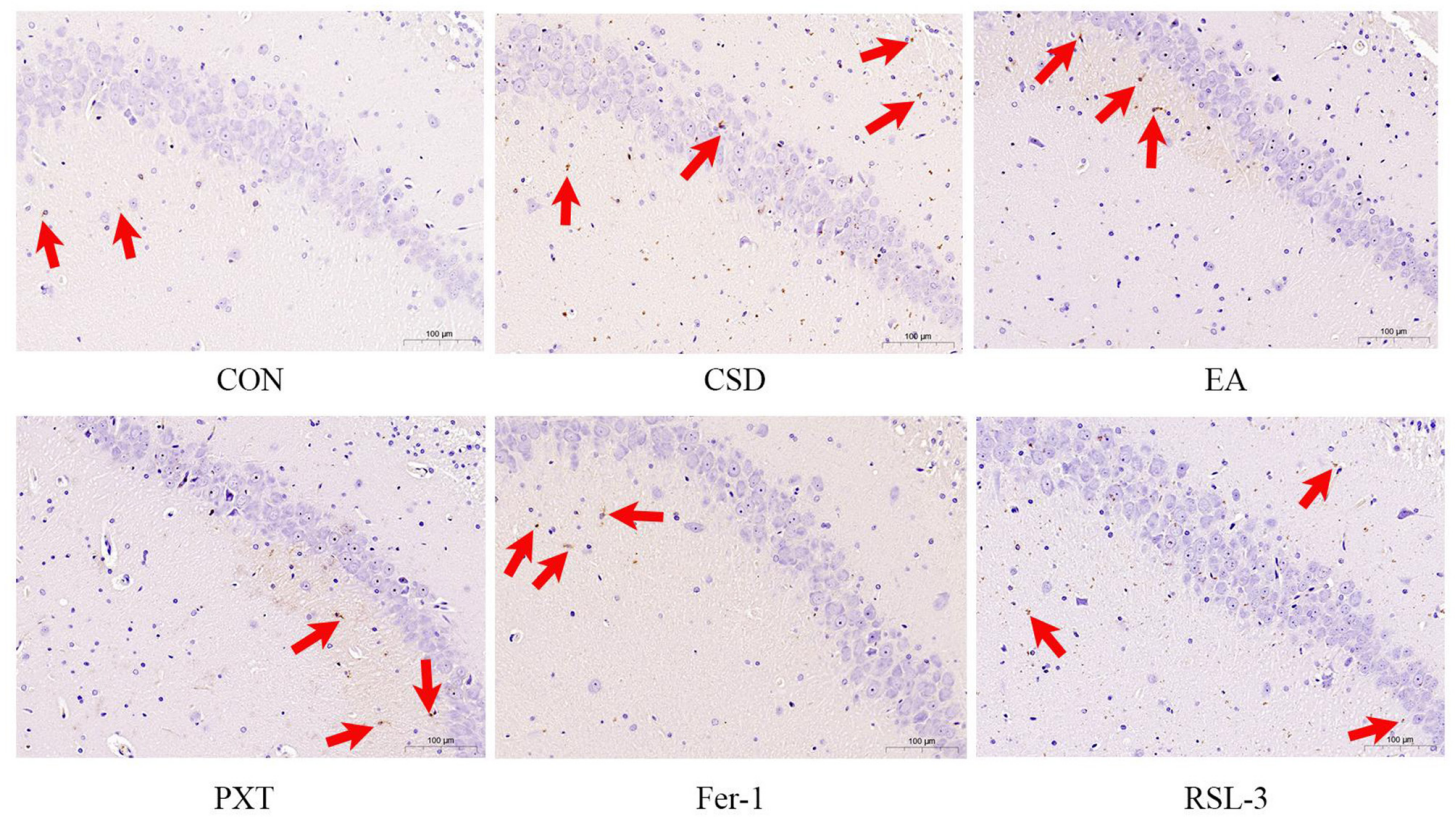
The iron deposition in the hippocampus of rats in each group is shown in the figure (scale bar: 100 μm, *n* = 3).

In the CON group, the hippocampal tissue of rats displayed a uniform light-blue background with no obvious iron deposition. The neurons exhibited normal morphology, with tightly packed cells and clearly visible nuclei. In contrast, the CSD group showed neuronal necrosis, nuclear shrinkage, and abundant yellow-brown iron deposits in the hippocampal tissue, indicating significant hippocampal iron accumulation induced by chronic sleep deprivation (as shown in Figure 5).

**Figure 6:**
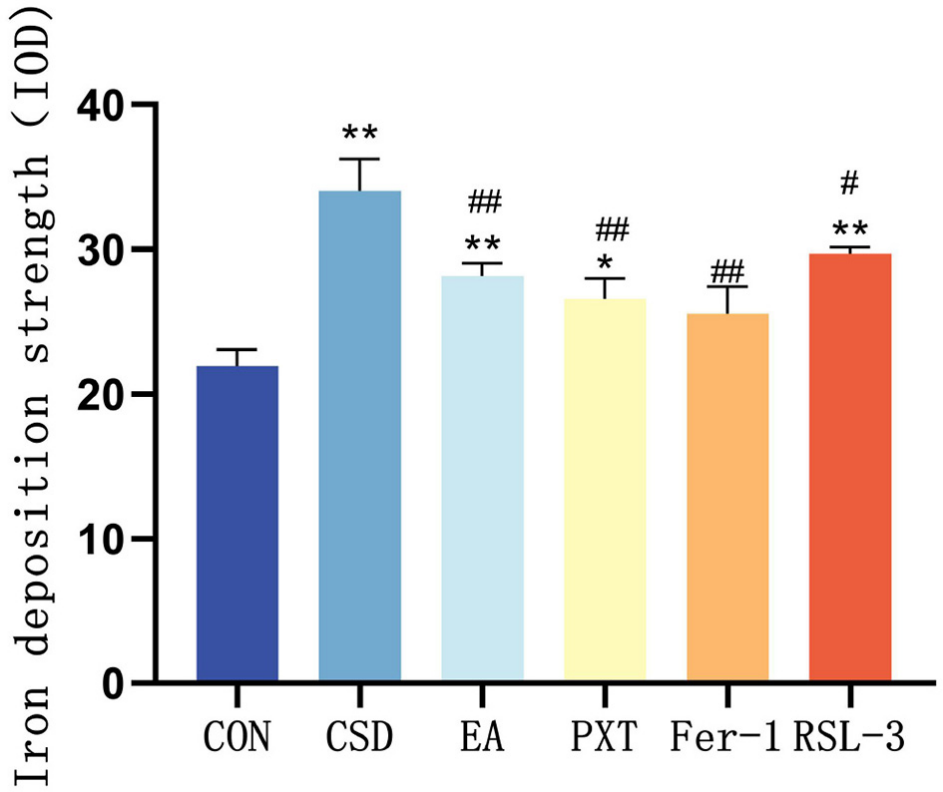
Quantitative comparison of hippocampal iron deposition based on IOD values (*n* = 3 per group.*P < 0.05, **P < 0.01 compared with the CON group; ^#^*P* < 0.05, ^##^*P* < 0.01 compared with the CSD group.

Quantitative analysis of iron deposition (IOD value) further confirmed these observations. Compared with the CON group, the CSD group exhibited a significantly higher IOD value (*P* < 0.01), suggesting pronounced hippocampal iron overload. Compared with the CSD group, the EA, PXT, and Fer-1 groups all showed significantly reduced iron deposition levels (*P* < 0.01), indicating that these interventions effectively alleviated CSD-induced disturbances in iron metabolism. Among them, the Fer-1 group exhibited the greatest reduction, consistent with its direct ferroptosis-inhibitory mechanism, while the EA group showed a comparable degree of reduction, suggesting that electroacupuncture can indirectly regulate iron metabolism. The RSL-3 group exhibited a slight decrease in IOD compared with the CSD group (*P* < 0.05), indicating that RSL-3 could induce ferroptosis and aggravate iron accumulation, although partial recovery was observed under electroacupuncture intervention (as shown in Figure 6).

### 3.3 Comparison of mitochondrial autophagy in hippocampal neurons of rats in each group

**Figure 7:**
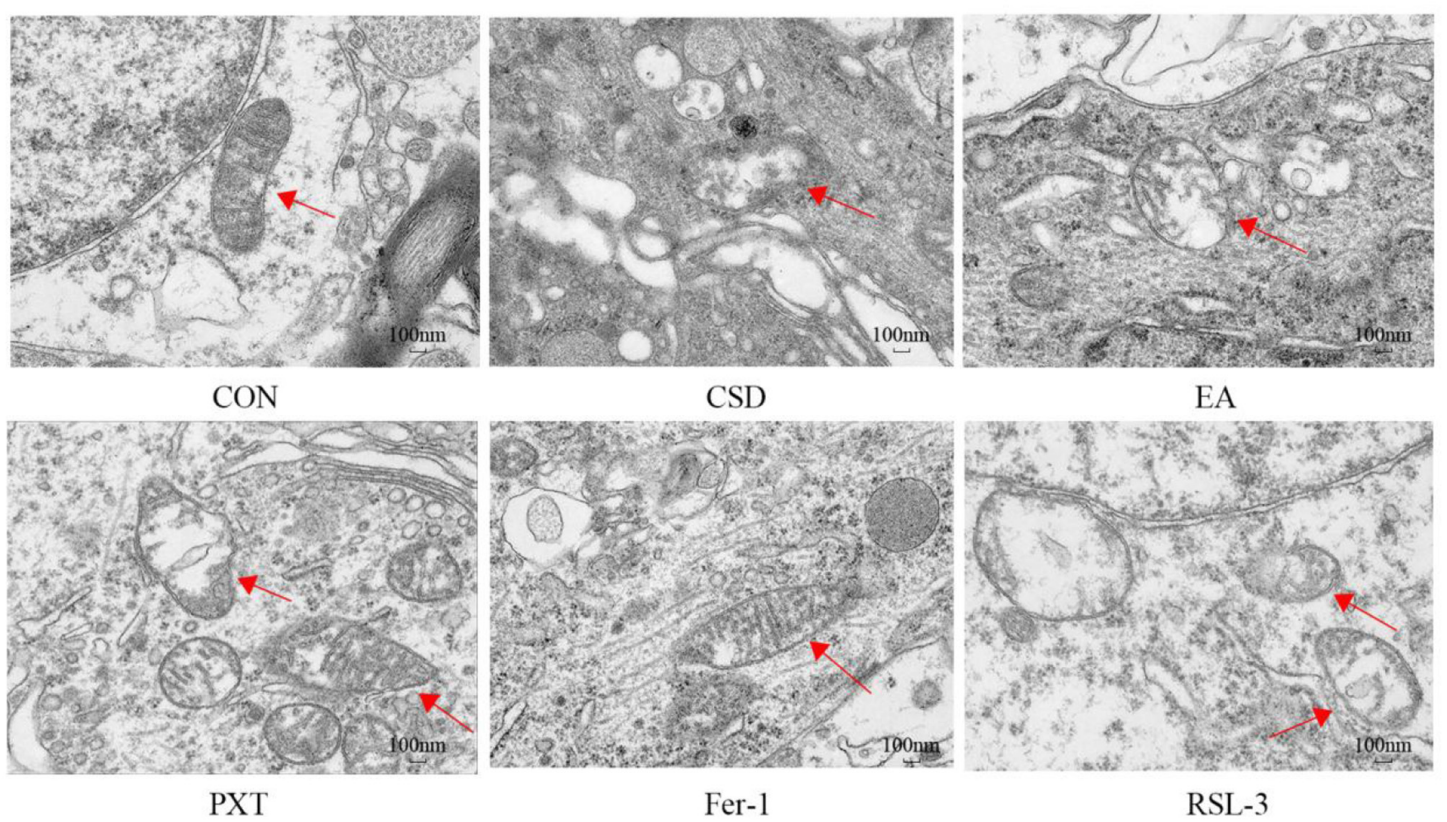
TEM imaging of the mitochondrial ultrastructure and autophagy in hippocampal neurons in rats (scale bar: 100 nm, *n* = 3).

The CON group had a normal number of mitochondria in the hippocampal neurons, with intact double membranes, normal mitochondrial crista morphology, and clear outlines. In contrast to the CON group, the CSD group showcased hippocampal neurons with mitochondria that were torn, bloated, and misshapen, featuring a thicker membrane, hazy edges, and several breaches. The mitochondrial cristae were notably diminished or completely vanished. Compared to the CSD group, the mitochondrial morphology in hippocampal neurons of the EA, PXT, and Fer-1 groups was relatively normal, with more intact double membranes and clearer mitochondrial cristae. Compared to the CSD group, the slight reduction in mitochondrial cristae of the EA, PXT, and Fer-1 groups was observed, and autophagic bodies were visible. The RSL-3 group had a small number of mitochondria with relatively intact structures, clear boundaries, and a normal membrane density. However, the RSL-3 group showed partial loss of mitochondrial cristae and blurred membrane demarcation (as shown in Figure 7).

### 3.4 Comparison of Fe^2+^, GSH, and MDA levels in hippocampal tissue of rats in each group

Compared to the CON group, the CSD group exhibited significantly increased levels of Fe^2+^ and MDA in hippocampal tissue (*P* < 0.01), and significantly decreased levels of GSH (*P* < 0.01). Compared to the CSD group, the EA, PXT, Fer-1, and RSL-3 groups had significantly decreased levels of Fe^2+^ and MDA in the hippocampus (*P* < 0.01), and significantly increased levels of GSH (*P* < 0.01).

**Figure 8:**
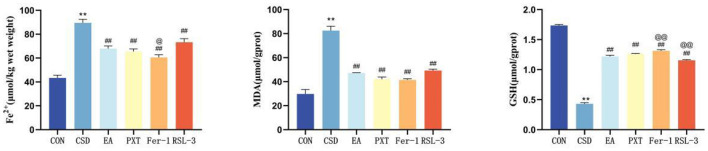
Levels of Fe^2+^, GSH, and MDA in the hippocampal tissue of rats in each group (*n* = 3). **P < 0.01 vs. CON group; ^##^*P* < 0.01 vs. CSD group; ^@^*P* < 0.05, ^@@^*P* < 0.01 vs. EA group.

Compared to the EA group, the Fer-1 group had significantly decreased Fe^2+^ levels in the hippocampus (*P* < 0.05) and significantly increased GSH levels (*P* < 0.01). The RSL-3 group had significantly decreased GSH levels in hippocampal tissue (*P* < 0.01). No statistically significant differences were found in the other comparisons (as shown in Figure 8 and Table 1).

**Table 1:** Levels of Fe^2+^, GSH, and MDA in hippocampal tissue of rats in each group (*n* = 3).

**Group**	**Fe^2+^ (μmol/kg wet weight)**	**MDA (μmol/g prot)**	**GSH (μmol/g prot)**
CON	43.36 ± 1.30	29.75 ± 2.15	1.74 ± 0.01
CSD	89.54 ± 1.70^**^	82.54 ± 2.08^**^	0.43 ± 0.01^**^
EA	67.92 ± 1.30^##^	47.21 ± 0.14^##^	1.22 ± 0.01^##^
PXT	65.47 ± 1.30^##^	41.97 ± 1.11^##^	1.26 ± 0.01^##^
Fer-1	60.55 ± 1.30^##@^	41.43 ± 0.59^##^	1.31 ± 0.01^##@@^
RSL-3	73.33 ± 1.70^##^	49.29 ± 0.61^##^	1.16 ± 0.01^##@@^

^**^P < 0.01 vs. CON group; ^##^*P* < 0.01 vs. CSD group; ^@^*P* < 0.05, ^@@^*P* < 0.01 vs. EA group.

### 3.5 Expression of ferroptosis-related proteins, circadian clock proteins, antioxidant system proteins, and lipid peroxidation-related proteins in the hippocampus of rats in each group

The levels of a number of proteins were examined in the hippocampal tissue of the rats in each group: the circadian clock proteins brain and muscle ARNT-like 1 (18) (BMAL1, also known as ARNTL) and circadian locomotor output cycles kaput (19) (CLOCK); the iron metabolism proteins ferritin heavy chain 1 (20) (FTH1) and ferritin light chain (21) (FTL); the antioxidant system proteins solute carrier family 7 member 11 (22) (SLC7A11) and glutathione peroxidase 4 (23) (GPX4), and the lipid peroxidation-related proteins Acyl-CoA synthetase long-chain family member 4 (24) (ACSL4), lysophosphatidylcholine acyltransferase 3 (25) (LPCAT3), and lysyl oxidase (26) (LOX). To facilitate the interpretation of molecular findings, the main biological functions of the detected proteins are briefly summarized below. BMAL1 (ARNTL): A core circadian transcription factor that regulates the rhythmic expression of genes related to metabolism, autophagy, and ferroptosis. CLOCK: Forms a heterodimeric complex with BMAL1 to drive the transcription of circadian clock-controlled genes. FTH1/FTL: The heavy and light chain subunits of the ferritin complex responsible for intracellular iron storage and regulation of iron homeostasis. SLC7A11: A key component of the cystine/glutamate antiporter system Xc^−^, involved in glutathione (GSH) synthesis and inhibition of lipid peroxidation. GPX4: A member of the glutathione peroxidase family and a crucial anti-ferroptotic enzyme that eliminates lipid peroxides. ACSL4: A member of the long-chain acyl-CoA synthetase family that catalyzes the acylation of polyunsaturated fatty acids (PUFAs), providing substrates for lipid peroxidation. LPCAT3: Promotes the remodeling of phospholipid membranes with polyunsaturated fatty acids and is closely associated with ferroptosis sensitivity. LOX: Lipoxygenase, which catalyzes the oxidation of polyunsaturated fatty acids to generate lipid peroxides, thereby promoting the ferroptosis process.

**Figure 9:**
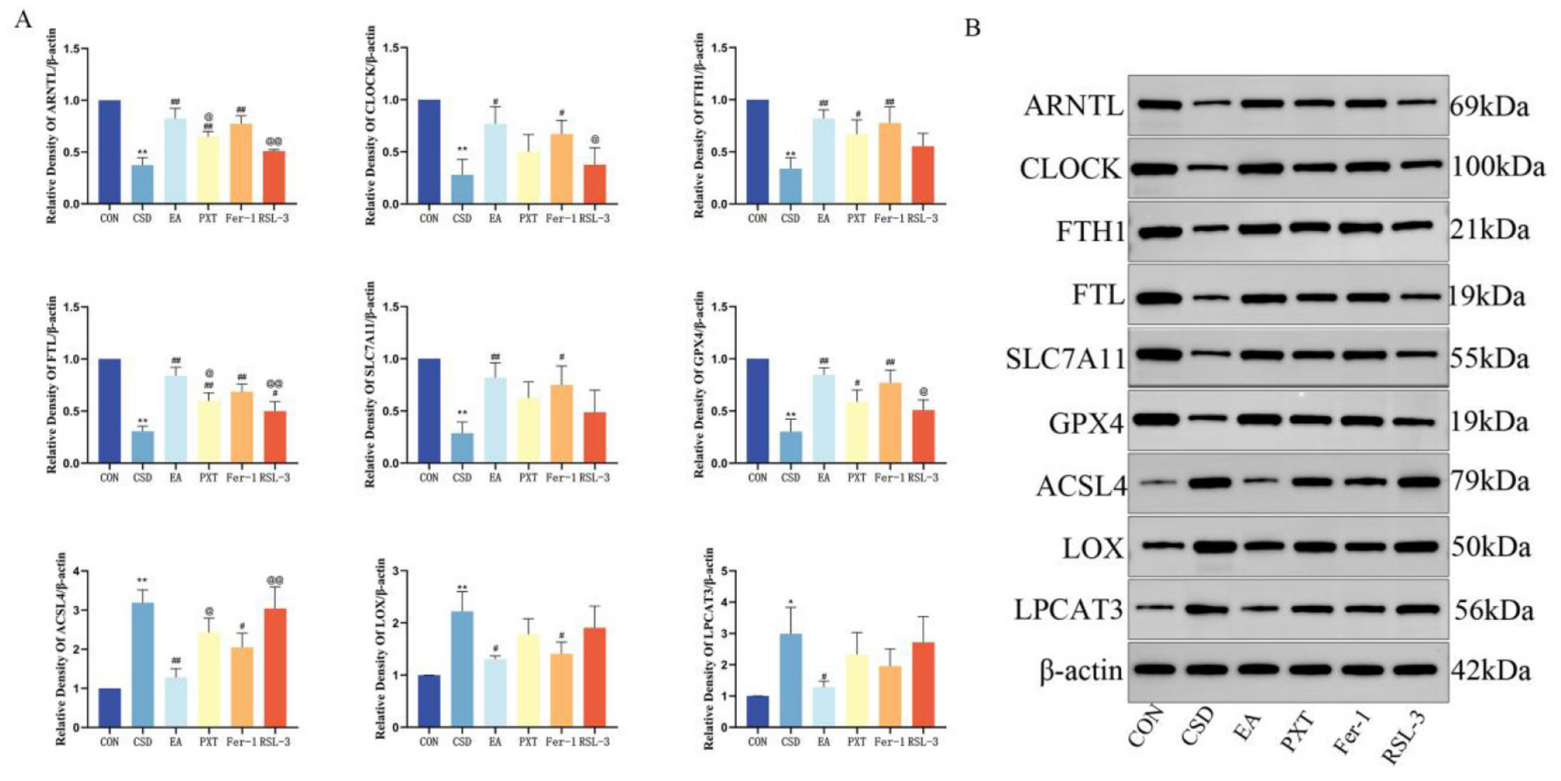
**(A)** Relative expression levels of target proteins. **(B)** Representative western blot bands (*n* = 3). *P < 0.05, **P < 0.01 vs. CON group; ^#^*P* < 0.05, ^##^*P* < 0.01 vs. CSD group; ^@^*P* < 0.05, ^@@^*P* < 0.01 vs. EA group.

Compared with the CON group, the levels of ARNTL, CLOCK, FTH1, FTL, SLC7A11, and GPX4 in the hippocampus of CSD rats were significantly decreased (*P* < 0.01), while the levels of ACSL4, LOX, and LPCAT3 were significantly increased (*P* < 0.01, *P* < 0.05). Compared with the CSD group, the EA, PXT, Fer-1, and RSL-3 groups had significantly increased levels of ARNTL, CLOCK, FTH1, FTL, SLC7A11, and GPX4 (*P* < 0.01, *P* < 0.05), and significantly decreased expression of ACSL4, LOX, and LPCAT3 (*P* < 0.01, *P* < 0.05). Compared with the EA group, ARNTL and FTL levels were significantly decreased in the PXT and RSL-3 groups (*P* < 0.01, *P* < 0.05, respectively), CLOCK and GPX4 levels were significantly decreased in the RSL-3 group (*P* < 0.05), and ACSL4 levels were significantly increased in both the PXT and RSL-3 groups (*P* < 0.01, *P* < 0.05, respectively). Other contrasts showed no statistical importance (as shown in Figure 9).

### 3.6 Confocal microscopy analysis of ARNTL and autophagosome (LC3) colocalization in the hippocampus of rats in each group

**Figure 10:**
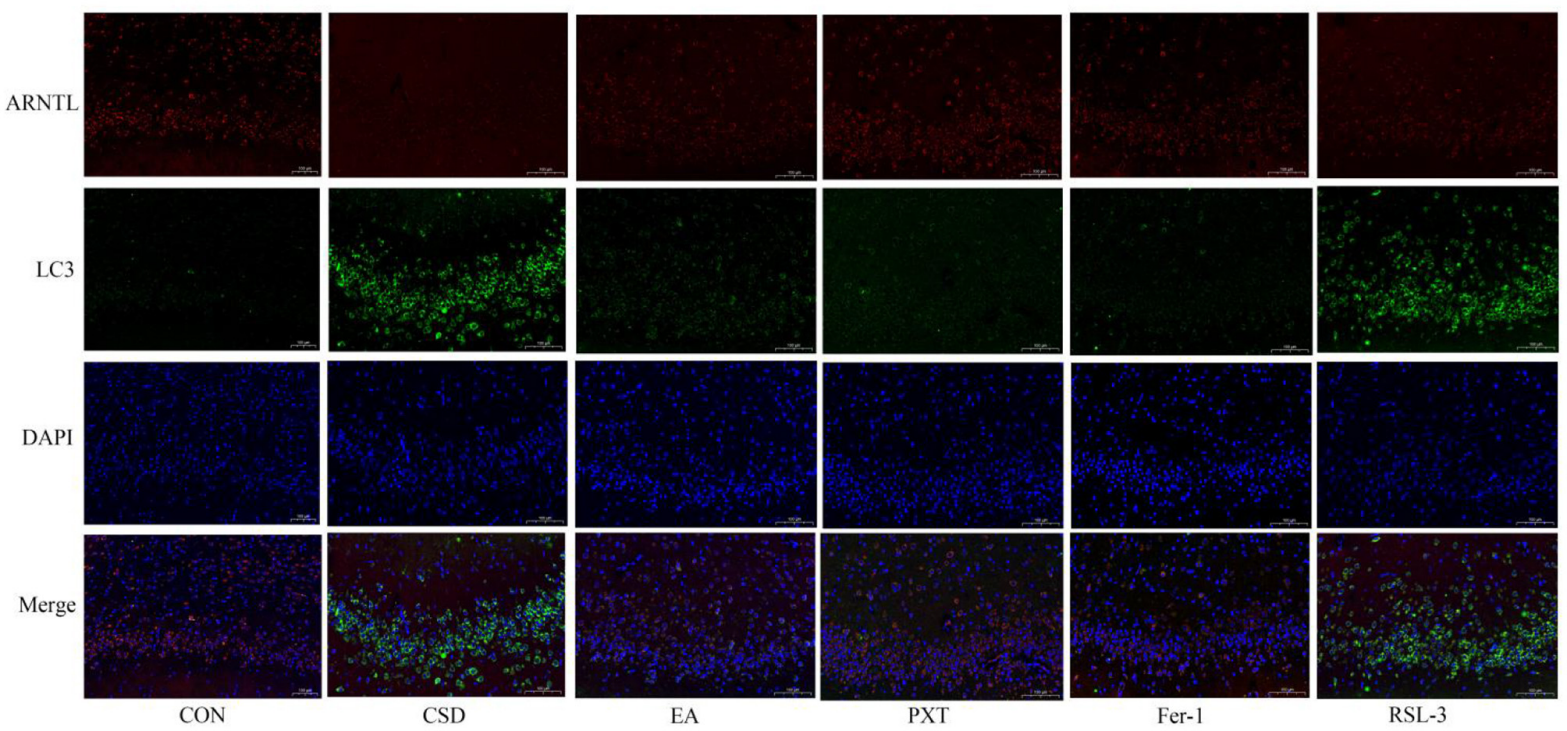
Colocalization of circadian clock autophagy protein ARNTL with autophagosomes in rat hippocampal tissue of each group (scale bar: 100 μm, *n* = 3).

**Figure 11:**
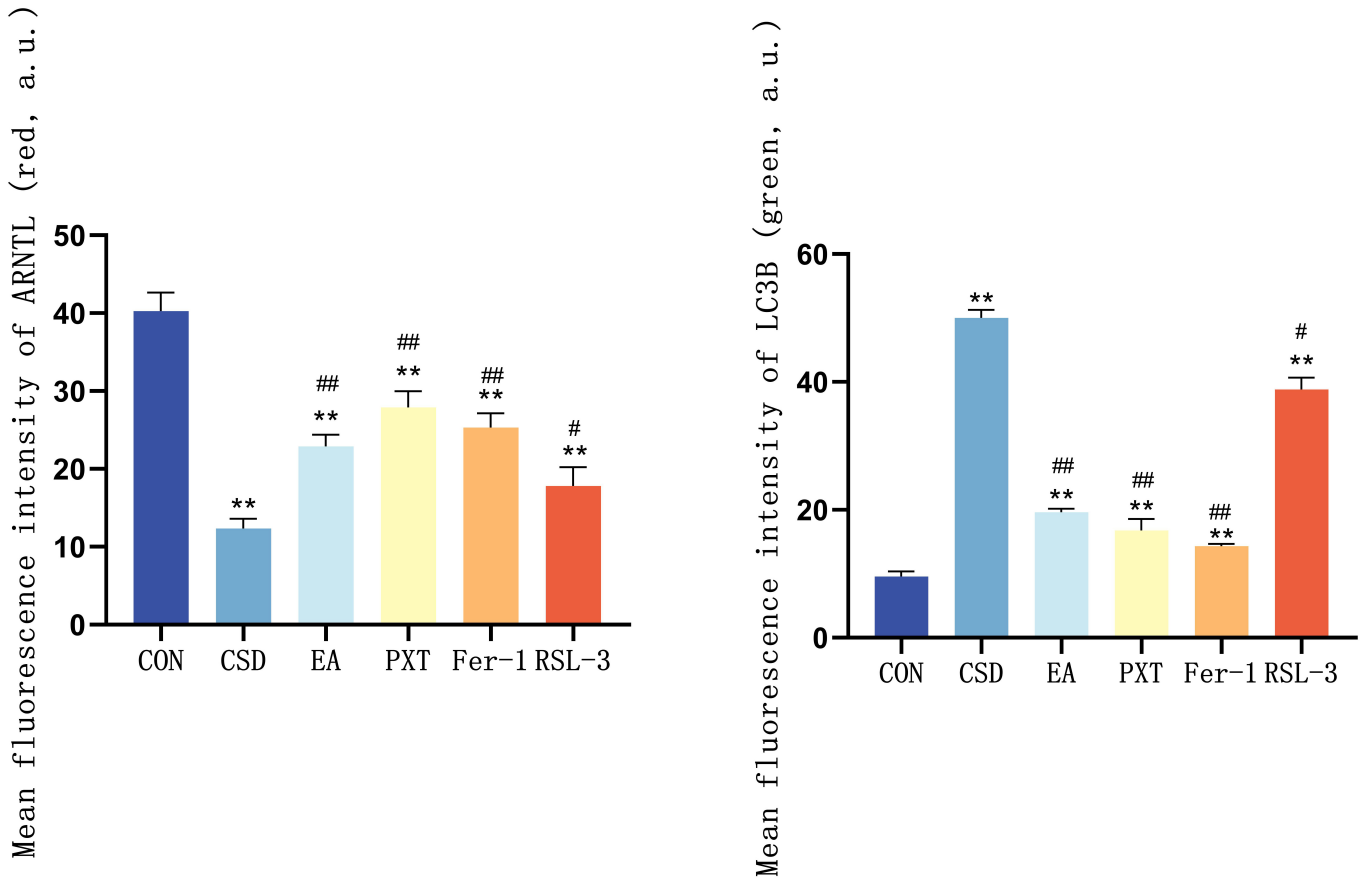
Quantification of ARNTL and LC3 fluorescence intensity in the hippocampus of rats from different groups (*n* = 3). **P < 0.01 vs. CON group; ^#^*P* < 0.05, ^##^*P* < 0.01 vs. CSD group.

In the CON group, ARNTL (red) fluorescence was strong, while LC3 (green) fluorescence was weak, indicating high ARNTL expression and low autophagic activity. In contrast, the CSD group showed reduced ARNTL and increased LC3 fluorescence, suggesting circadian rhythm suppression and autophagy overactivation. Compared with CSD, the EA, PXT, Fer-1, and RSL-3 groups exhibited partial recovery of ARNTL and reduced LC3 signals, indicating restoration of rhythm–autophagy balance (as shown in Figure 10). Quantitatively, ARNTL fluorescence intensity was significantly decreased and LC3 increased in the CSD, EA, PXT, Fer-1, and RSL-3 groups vs. CON (*P* < 0.01). Relative to CSD, EA, PXT, Fer-1, and RSL-3 significantly enhanced ARNTL and reduced LC3 fluorescence (*P* < 0.01, *P* < 0.05). EA produced effects comparable to PXT and Fer-1, suggesting that electroacupuncture effectively mitigates CSD-induced rhythm suppression and autophagy overactivation in the hippocampus (as shown in Figure 11).

## 4 Discussion

This study provides novel evidence that EA alleviates depression induced by CSD during adolescence, potentially through modulation of the circadian rhythm–autophagy–ferroptosis axis in the hippocampus. Specifically, EA was associated with the restoration of ARNTL/CLOCK expression, correlated with balanced autophagy activity, and accompanied by inhibition of ferroptosis-related lipid peroxidation, thereby contributing to improved neuronal survival and depressive-like behaviors. These findings expand the understanding of EA's neuroprotective mechanisms and highlight its system-level association with circadian and metabolic homeostasis. C SD is recognized as a pivotal risk factor in triggering depressive symptoms during the teenage years. This experimental design provides new insight into early prevention and individualized treatment strategies for adolescent depression related to chronic sleep deprivation. Existing research has largely examined neural impairments after depression develops, while early pathological mechanisms before onset remain understudied. Adverse environmental exposures during adolescence can induce long-lasting neurobiological changes, with hippocampal neuronal ferroptosis a key mediator of these long-term effects (27). Animal studies have shown that exposure to external stressors such as CSD during early life can lead to persistent disruptions in circadian gene expression patterns in the brain. These alterations endure throughout life, persisting into adulthood and ultimately taking the form of enduring depressive-like tendencies (28). In this study, a rodent sleep restriction device was employed to create a CSD rat model from childhood through adolescence. This method has been confirmed to effectively induce cognitive decline, increase anxiety-like behaviors, and produce depressive-like behaviors in rats (29). These data aligned with prior investigations, reinforcing the concept that adolescent CSD leads to adult depressive characteristics. Extending the span of modeling from childhood into the teenage years allowed for a broader, lifecycle perspective on the impact of CSD. This approach yielded a more comprehensive animal model, which is now crucial in delving into the lasting consequences of sleep deprivation during the early years on various neuropsychiatric issues. Compared to rats in the CON group, rats in the CSD group exhibited significant decreases in sucrose preference, increased delayed feeding times, decreased total distances traveled in the open field, reduced total number of grid crossings, decreased distances traveled in the central area, reduced time spent in the central area, increased immobility time in the forced swim test, and decreased struggling time on PND 42. These depressive-like behaviors persisted into adulthood. Interventions conducted between PND 42 and PND 64 showed that electroacupuncture significantly improved the decreased exploratory, anhedonic-like, and despair-like behaviors induced by CSD. Other intervention groups also showed some recovery of the behavioral indices. On PND 68, the EA group's sucrose preference and immobility time in the forced swim test showed no significant differences compared to those of the CSD group; however, on PND 93, the EA group showed significant differences in sucrose preference and immobility time. On PND 90, which corresponds to humans over 20 years old, the behavioral improvements were even more significant, indicating that acupuncture alleviated depressive-like behaviors induced by CSD from childhood to adolescence and treated depression in adulthood. The findings indicated that early CSD resulted in the development of depression in adulthood, and that the timing of electroacupuncture intervention, administered after sleep deprivation but before disease onset, could reverse depression caused by early CSD, reflecting the traditional Chinese medicine concept of “treating the disease before it manifests.” Comparative analysis among intervention groups revealed distinct yet complementary mechanisms. EA and PXT both improved depressive-like behaviors but acted through different pathways: PXT primarily enhanced serotonergic neurotransmission, whereas EA exerted multi-level regulation by restoring circadian rhythm, rebalancing autophagy, and suppressing ferroptosis. Fer-1, as a direct ferroptosis inhibitor, produced the most rapid reduction in hippocampal iron overload, confirming ferroptosis as a key contributor to CSD-induced pathology. Notably, in the RSL-3 group, EA partially reversed RSL-3–induced ferroptosis activation, suggesting that EA can counteract chemically triggered ferroptotic damage through upstream circadian–autophagic modulation.

Circadian rhythm disruption is a key internal mechanism linking CSD to the development of depression in adulthood. The core regulatory gene *ARNTL* reduces neuronal ferroptosis by inhibiting hypoxia-inducible factor EGLN expression and upregulating HIF1A activity. This molecular pathway was shown to form the biological foundation by which adolescent CSD resulted in depression in adulthood (30). This matched our results, showing reduced circadian rhythm protein levels in the hippocampi of CSD rats. Mechanistically, EA was associated with the modulation of the circadian rhythm–autophagy–ferroptosis axis disrupted by CSD. Upregulation of ARNTL/CLOCK coincided with rhythmic transcriptional changes in autophagy-related genes, normalization of LC3 activity, and a reduction in excessive autophagic flux. These alterations were accompanied by attenuated lipid peroxidation, characterized by increased GPX4 and SLC7A11 expression and decreased ACSL4/LPCAT3-mediated ferroptotic sensitivity. Thus, EA may indirectly influence ferroptosis through its association with upstream circadian–autophagy interactions. Circadian rhythm disruption has been increasingly recognized to drive ferroptosis by altering autophagy homeostasis and iron metabolism. This rhythm–autophagy–ferroptosis interplay forms the mechanistic foundation underlying CSD-induced neuronal injury. However, we acknowledge that the current evidence primarily demonstrates a strong correlation between EA-induced modulation of circadian rhythm, autophagy, and ferroptosis suppression, rather than a confirmed causal relationship. The data support the concept that circadian clock regulation may participate in autophagy–ferroptosis crosstalk, but do not conclusively establish “clock-controlled autophagy” as the dominant mechanism. Future functional studies—such as genetic knockdown or pharmacological inhibition of ARNTL/CLOCK—will be essential to verify whether EA's neuroprotective and antidepressant effects are dependent on circadian gene activity. Nevertheless, our results align with previous high-impact studies (31), showing that EA modulates circadian signaling, autophagy, and ferroptosis-related oxidative stress, providing consistent mechanistic evidence across independent models. Collectively, while our study cannot claim direct causality, the converging behavioral, molecular, and ultrastructural findings strongly support that EA exerts systemic regulation of the circadian–autophagy–ferroptosis network, which may underlie its antidepressant and neuroprotective effects. Ferroptosis involves iron-driven buildup of peroxidized lipids, which ultimately causes cell death. Contrasting typical cell-death patterns, ferroptosis features unique morphological and biochemical traits (32). Ferroptotic cells often show marked mitochondrial contraction, elevated membrane density, and diminished, degenerated, or absent mitochondrial cristae. Although the cell nuclei of ferroptotic cells remain normal in size, they lacks chromatin condensation (33). Consistently, the hippocampal tissue of CSD rats in our study exhibited neuronal necrosis, nuclear shrinkage, and extensive iron deposition that was significantly abnormal. TEM results further confirmed that hippocampal neurons of CSD rats had significantly damaged mitochondria, with shrinkage, reduced cristae, and ruptured membranes, consistent with the mitochondrial swelling or shrinkage and fewer or absent cristae observed in depression patients (34). In depression models, similar ferroptosis-related changes have been observed, including reduced mitochondrial plasticity, a decreased mitochondrial volume, and fewer mitochondrial cristae (35). After intervention, the hippocampal neurons in each group of rats showed dense morphological arrangements, and the mitochondrial ultrastructure returned to normal.

Ferroptosis is also associated with abnormal iron metabolism. In ferroptosis, the intracellular free Fe^2+^ level is significantly elevated, and as Fe^3+^ enters the cell through transferrin receptor 1 and reduced to Fe^2+^, forming an unstable iron pool. This pool participates in the Fenton reaction, producing large amounts of hydroxyl radicals that promote lipid peroxidation (36). Fer-1 and RSL-3 are compounds that inhibit and induce ferroptosis in cells, respectively, through mechanisms related to intracellular iron metabolism, lipid peroxidation processes, and antioxidant activities. Consistent with the findings of Ma PW (37) and colleagues, we observed that the CSD group exhibited increased Fe^2+^ levels, downregulated FTH1 and FTL protein levels, and decreased ARNTL fluorescence signals with a corresponding increase in LC3 fluorescence signals. This suggested that electroacupuncture alleviated the CSD-induced inhibition of ARNTL expression and excessive autophagy activation, with similar improvements observed in the other intervention groups. GPX4 is an essential antioxidant enzyme within the cell, responsible for reducing lipid peroxides and maintaining cell membrane stability. Two key features of ferroptosis are lipid peroxidation and the loss of GPX4 activity (38). Additionally, MDA, a biomarker of oxidative stress, modifies biomolecules by forming covalent electrophilic adducts, which promotes the occurrence of ferroptosis and other disease processes (39). Aligned with current findings, the CSD group showed reduced hippocampal GSH and diminished related protein expression in rats. The expression of lipid oxidative stress-related proteins was upregulated, and the MDA level increased. In comparison with the CSD group, the intervention groups showed significant improvement in these pathological markers. An imbalance in ROS levels can prevent the body from effectively clearing lipid peroxides, which further promotes lipid peroxidation. In the presence of LOX, lipids undergo ferroptosis (40). Moreover, overexpression of SLC7A11 increased cysteine uptake and enhanced GSH synthesis, which in turn inhibited ferroptosis (41). ACSL4 is also a key substrate in lipid peroxidation during ferroptosis. Ferroptosis is dependent on lipid peroxidation, and electroacupuncture can regulate iron metabolism pathways, reduce iron accumulation, and thereby lower the risk of lipid peroxidation (42). Furthermore, PXT, a commonly used medication for depression, effectively improves sleep disturbances in patients and alleviates depressive symptoms to some extent (43). Consistent with the research by Xu Y (44), in the present study, the levels of ACSL4, LOX, and LPCAT3 in the CSD group were significantly reduced; after electroacupuncture intervention, the levels were restored to normal. Electroacupuncture also reversed the effects of RSL-3 and produced positive therapeutic outcomes. The functional roles of the proteins examined—particularly ARNTL, GPX4, SLC7A11, and ACSL4—collectively reflect the circadian–autophagy–ferroptosis axis targeted by EA treatment. ARNTL regulates rhythmic transcription of autophagy- and metabolism-related genes, while GPX4 and SLC7A11 inhibit lipid peroxidation, and ACSL4/LPCAT3 promote ferroptosis sensitivity.

Based on adolescent physiological characteristics—such as hormonal fluctuation, neural plasticity, and circadian sensitivity—clinical EA should adopt gentle, sustained stimulation (0.5–1 mA, 2–15 Hz, 20–30 min) targeting GV20 (Baihui), EX-HN3 (Yintang), LI4 (Hegu), and LR3 (Taichong) to stabilize emotion and improve sleep regulation. EA can be combined with behavioral or pharmacological therapy to enhance clinical efficacy. For example, combining EA with low-dose SSRIs (e.g., paroxetine) improves antidepressant response while reducing side effects (45). Overall, EA represents a safe, non-pharmacological, and sustainable therapeutic approach particularly suitable for adolescents, aligning with the TCM principle of “treating the disease before it manifests.”

However, we acknowledge that the current evidence primarily demonstrates a strong correlation between EA-induced modulation of circadian rhythm, autophagy, and ferroptosis suppression, rather than a confirmed causal relationship. The data support the concept that circadian clock regulation may participate in autophagy–ferroptosis crosstalk, but do not conclusively establish “clock-controlled autophagy” as the dominant mechanism. Future functional studies—such as genetic knockdown or pharmacological inhibition of ARNTL/CLOCK—will be essential to verify whether EA's neuroprotective and antidepressant effects are dependent on circadian gene activity. In addition, EA may exert antidepressant-like and neuroprotective effects through multiple complementary mechanisms beyond ferroptosis inhibition, such as anti-inflammatory regulation, antioxidant defense, autophagy balance, synaptic plasticity enhancement, and modulation of hypothalamic–pituitary–adrenal and circadian systems. These multifactorial actions may produce therapeutic effects similar to conventional antidepressants.

**Figure 12:**
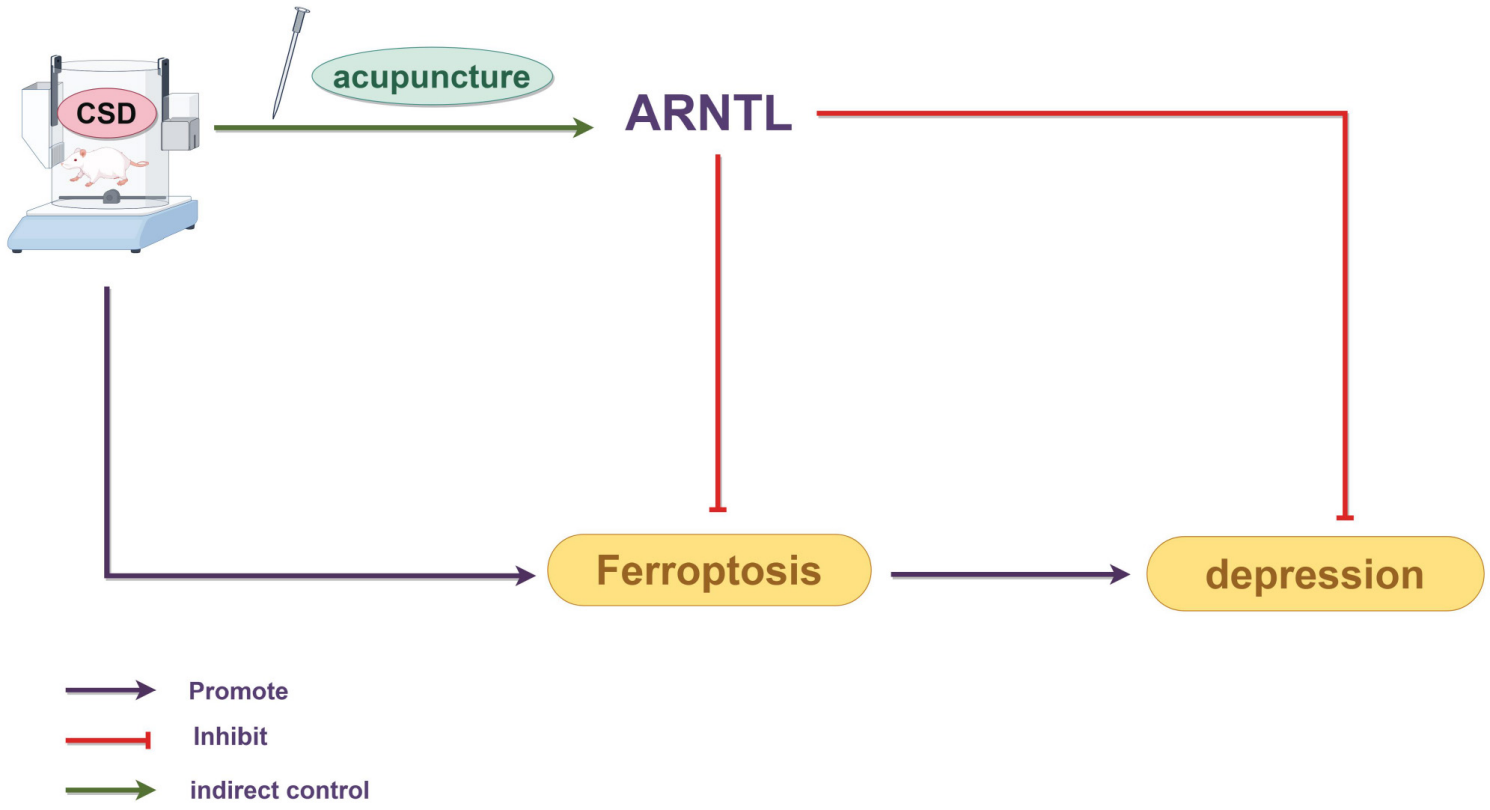
Mechanism by which electroacupuncture regulates circadian autophagy to inhibit hippocampal neuronal ferroptosis and alleviate depression-like behaviors induced by early chronic sleep deprivation in adulthood.

At PND65, the RSL-3 group showed significant improvements in struggling/swimming time and open-field activity compared with the CSD group (*P* < 0.01), while no significant differences were observed between RSL-3 and EA. By PND90, RSL-3-treated rats exhibited higher sucrose preference, longer swimming/struggling time, and greater open-field locomotion than CSD (*P* < 0.01), but a shorter central-zone distance compared with EA (*P* < 0.05). This behavioral pattern—RSL-3 differing from CSD but remaining comparable to EA at early stages—likely reflects a time-dependent interplay between ferroptosis induction and the multi-target protective mechanisms of EA. Although RSL-3 activates ferroptosis by inhibiting GPX4, it does not completely eliminate endogenous protective processes such as antioxidant and autophagic responses. Meanwhile, EA exerts broader and longer-lasting actions beyond ferroptosis inhibition, including anti-inflammatory, antioxidant, autophagy-regulatory, circadian-stabilizing, and synaptic-plasticity–enhancing effects. These combined mechanisms may sustain behavioral recovery and account for the partial overlap and subsequent divergence between RSL-3 and EA groups over time. However, since the present study focused primarily on ferroptosis, the potential contributions of these alternative pathways were not investigated, which represents an important limitation of this work. Nevertheless, our results align with previous high-impact studies (46), showing that EA modulates circadian signaling, autophagy, and ferroptosis-related oxidative stress, providing consistent mechanistic evidence across independent models. Collectively, while our study cannot claim direct causality, the converging behavioral, molecular, and ultrastructural findings strongly support that EA exerts systemic regulation of the circadian–autophagy–ferroptosis network, which may underlie its antidepressant and neuroprotective effects. The mechanism diagram of this article is shown in Figure 12.

This study revealed a novel mechanism by which electroacupuncture regulates biological clock–dependent autophagy to inhibit ferroptosis, thereby alleviating depression and providing new insights for clinical therapy. However, the regulatory network of ferroptosis is highly complex, and future research should further explore whether electroacupuncture exerts its effects through additional mechanisms, such as oxidative stress, neuroinflammation, or neurotransmitter regulation across different brain regions. In particular, subsequent studies could employ genetic knockdown or knockout models targeting key circadian genes (e.g., ARNTL or CLOCK) to directly verify whether electroacupuncture's neuroprotective and antidepressant effects are dependent on circadian rhythm regulation. Furthermore, gender differences—such as hormonal fluctuations—may influence experimental outcomes; thus, future investigations should include female rats to assess the generalizability of these findings. Ultimately, well-designed clinical trials are warranted to evaluate the long-term efficacy and safety of electroacupuncture for depression, facilitating its translation into clinical application.

## Data Availability

The raw data supporting the conclusions of this article will be made available by the authors, without undue reservation.
