# Supplementary material for: Mechanistic study on electroacupuncture-regulated circadian autophagy for inhibiting ferroptosis in hippocampal neurons and alleviating depression-like behaviors in adulthood induced by early chronic sleep deprivation
Source: Front Neurol. 2025 Nov 7;16:1680606. doi: 10.3389/fneur.2025.1680606 (PMC12621143; doi:10.3389/fneur.2025.1680606)

Supplementary Material

**Supplement the original image of the WB.**

The first experiment

1.Internal reference protein-ACTIN


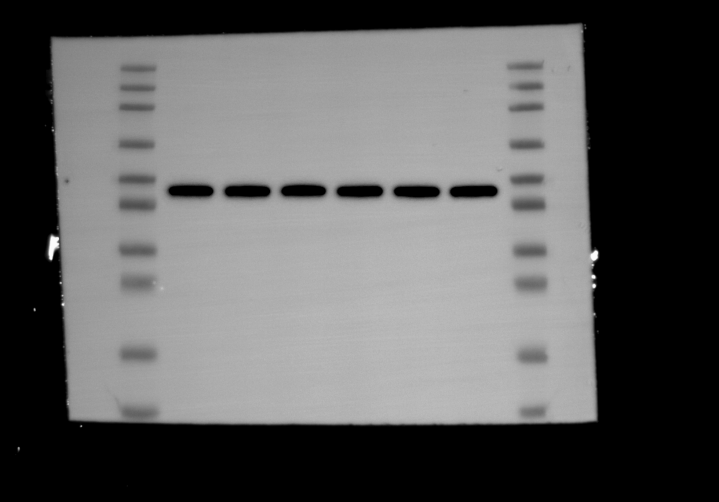


2.Interest protein-ARNTL


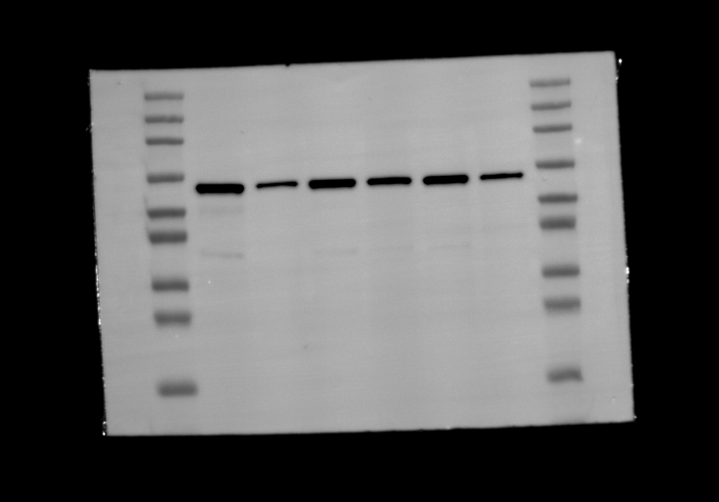


3.Interest protein-CLOCK


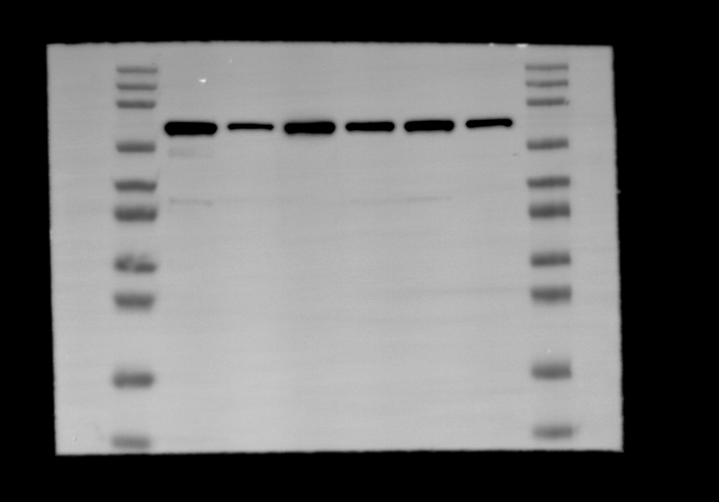


4.Interest protein-FTH1


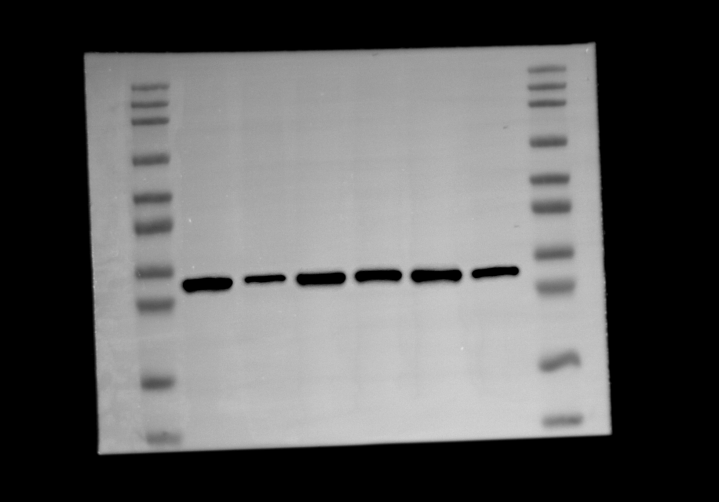


5.Interest protein-FTL


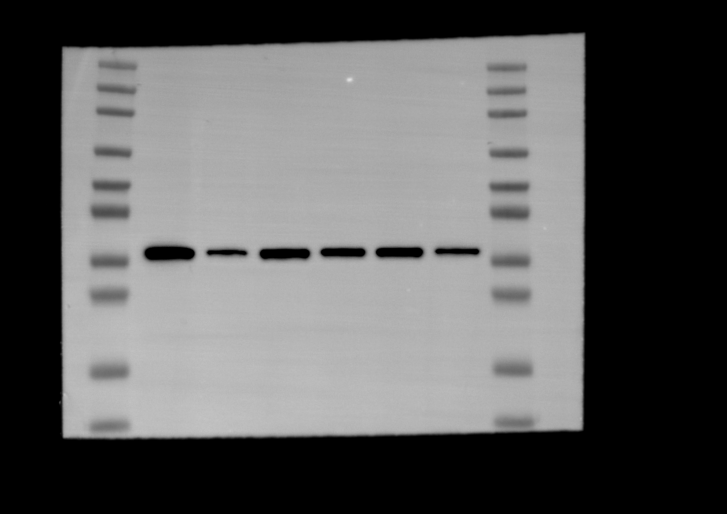


6.Interest protein-SLC7A11


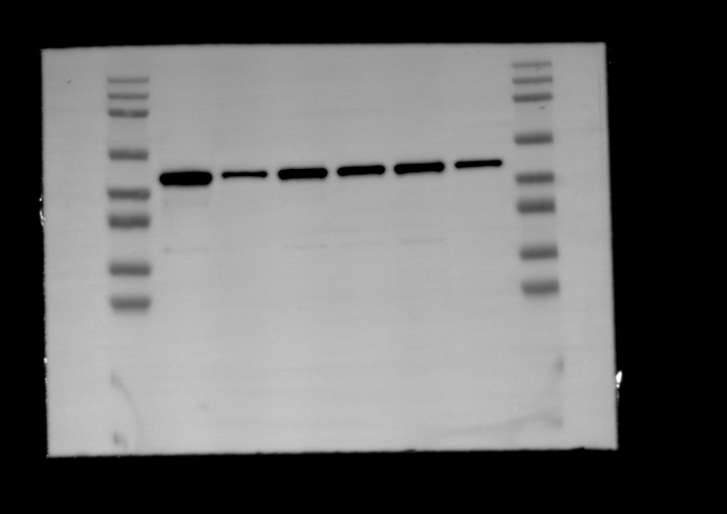


7.Interest protein-GPX4


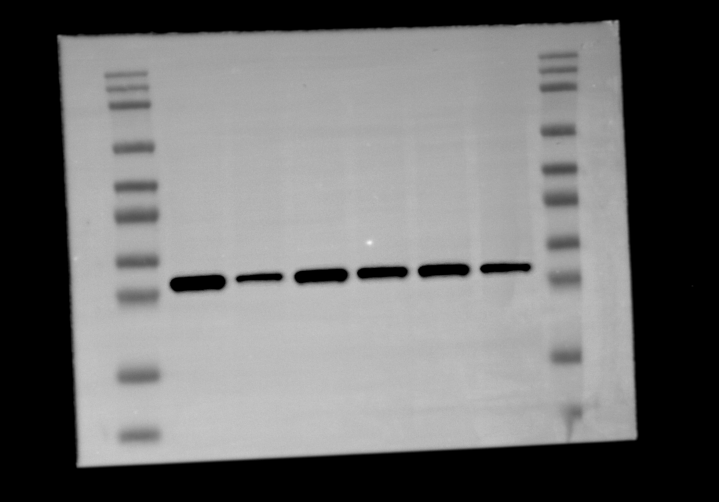


8.Interest protein-ACSL4


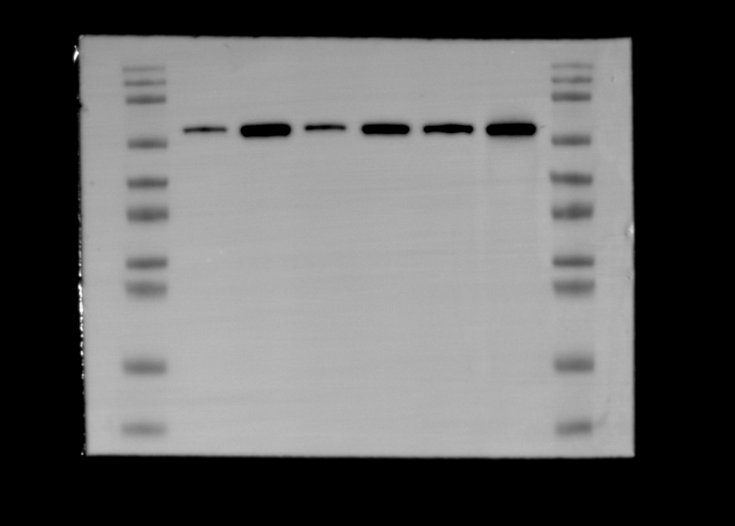


9.Interest protein-LOX


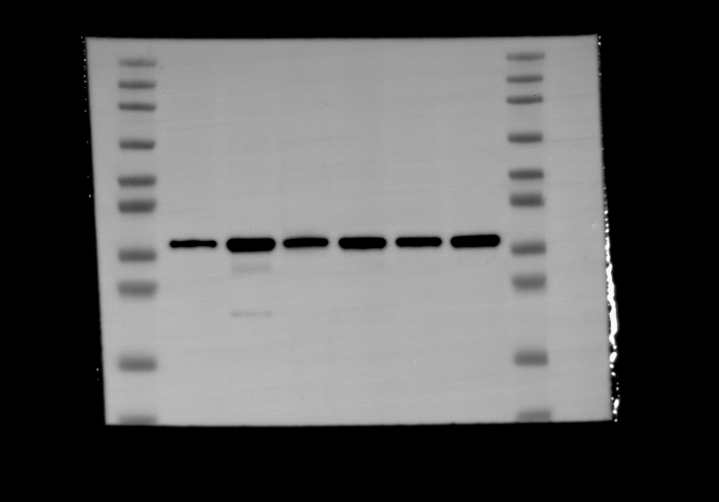


10.Interest protein-LPCAT3


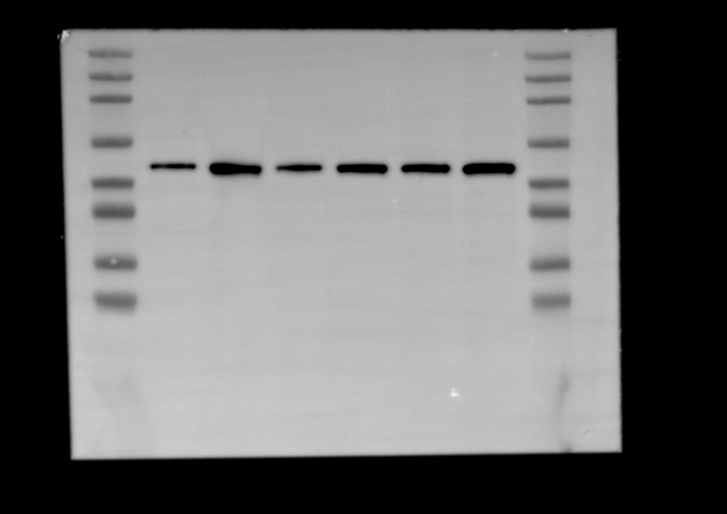


The second experiment

1.Internal reference protein-ACTIN


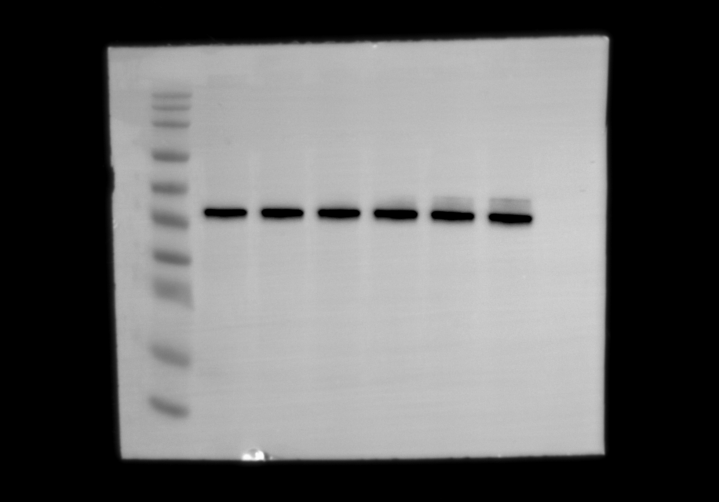


2.Interest protein-ARNTL


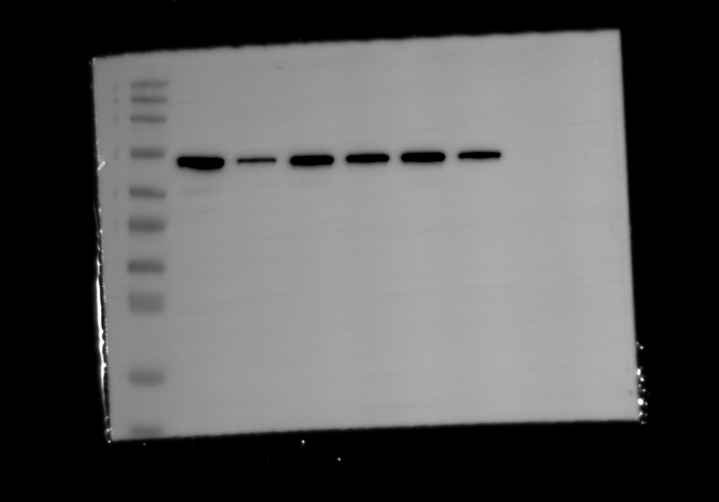


3.Interest protein-CLOCK


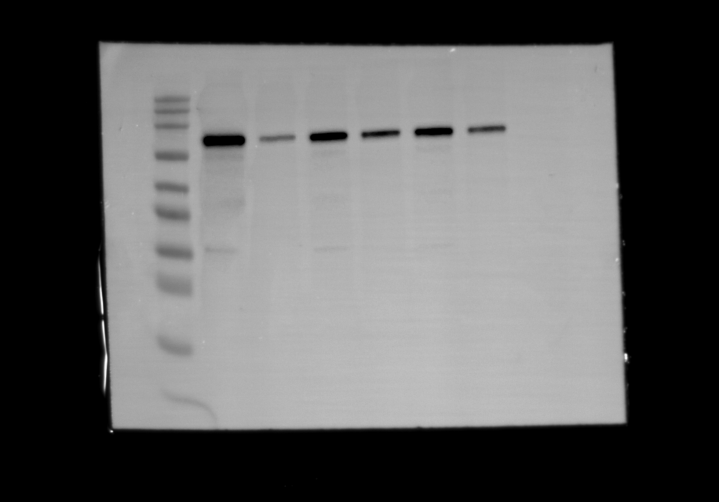


4.Interest protein-FTH1


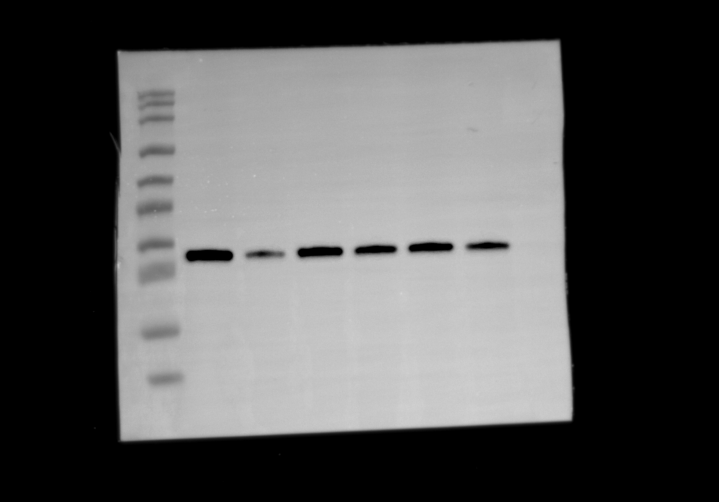


5.Interest protein-FTL


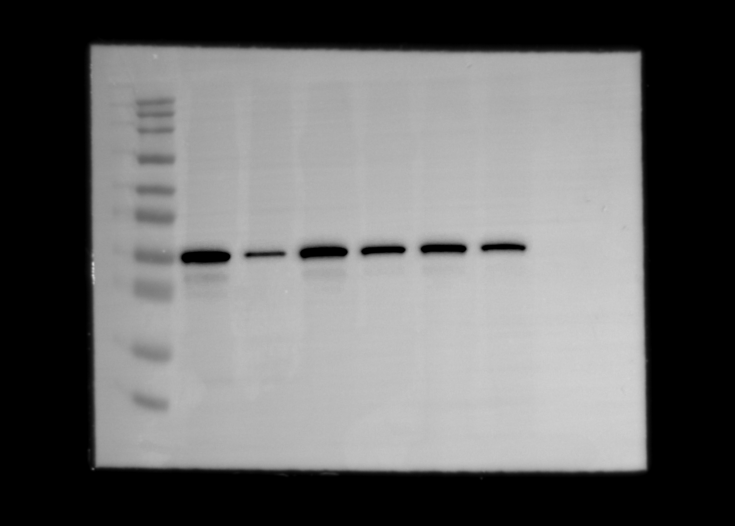


6.Interest protein-SLC7A11


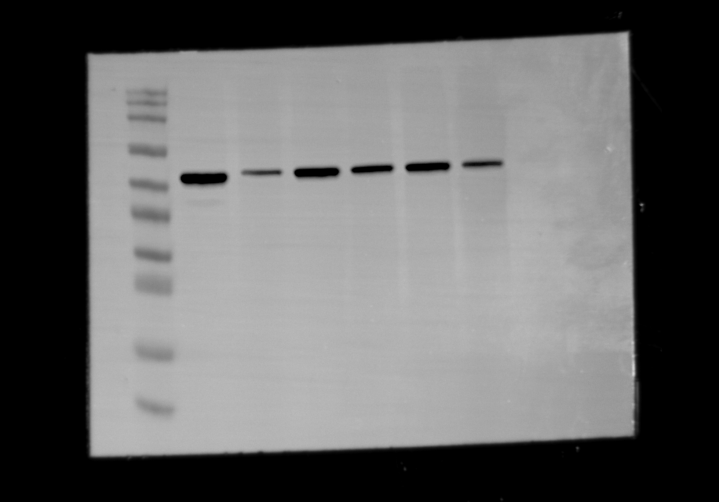


7.Interest protein-GPX4


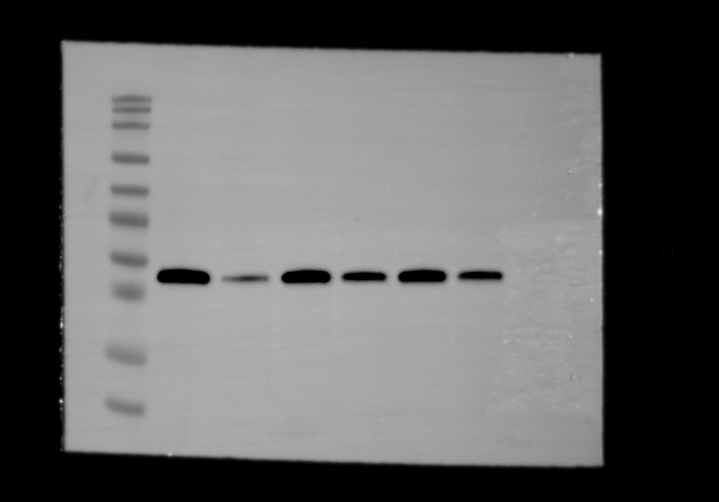


8.Interest protein-ACSL4


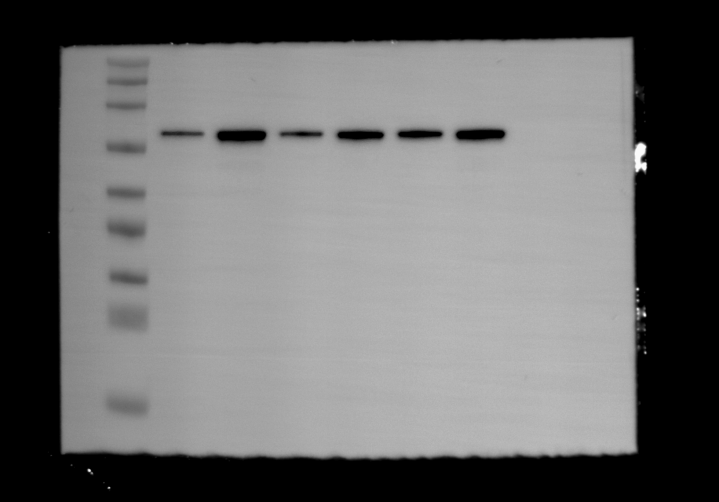


9.Interest protein-LOX


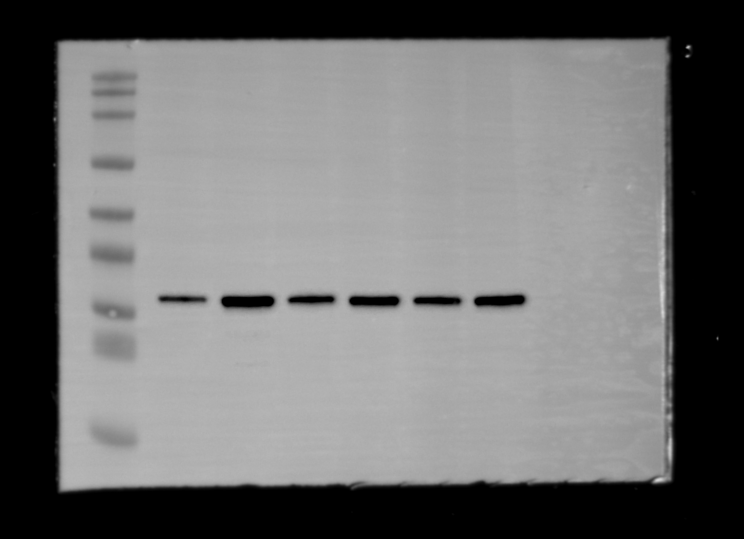


10.Interest protein-LPCAT3


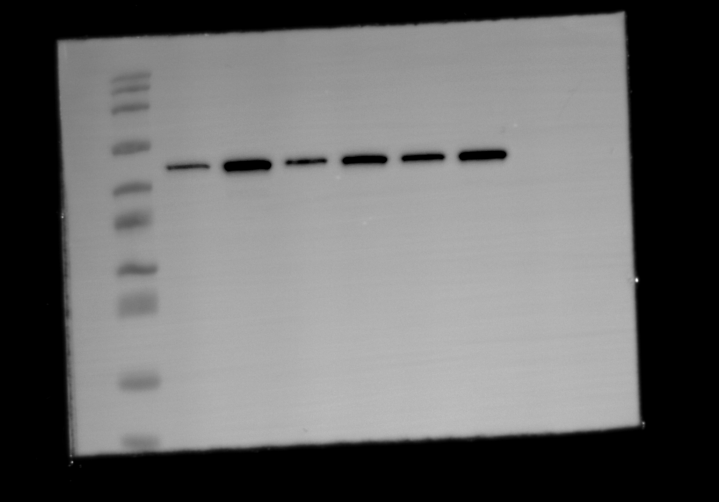


The third experiment

1.Internal reference protein-ACTIN


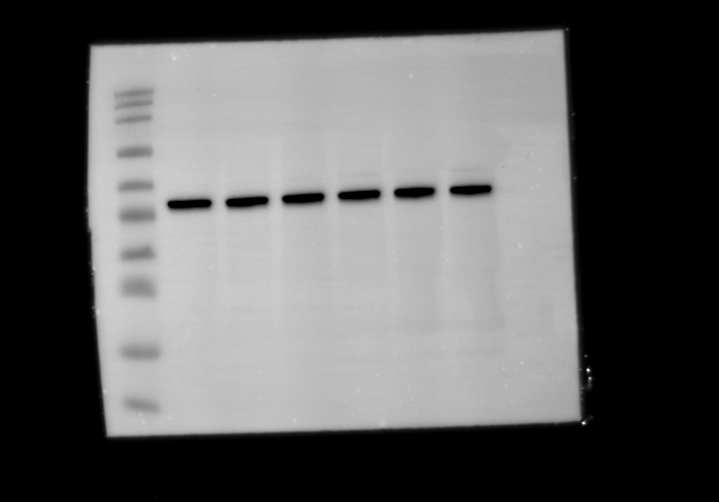


2.Interest protein-ARNTL


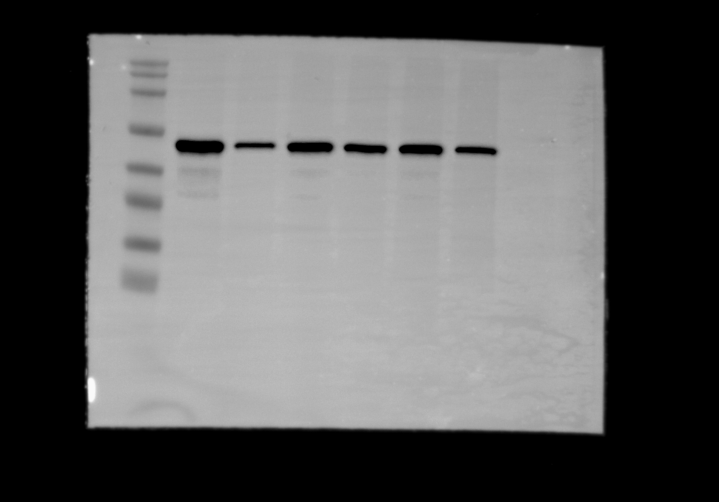


3.Interest protein-CLOCK


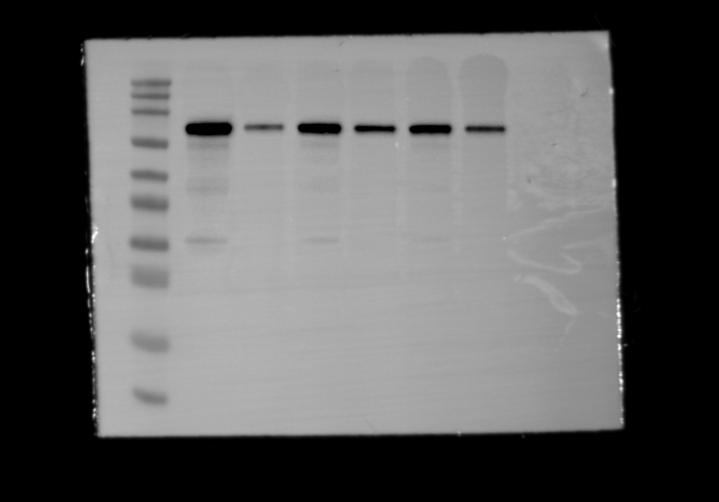


4.Interest protein-FTH1


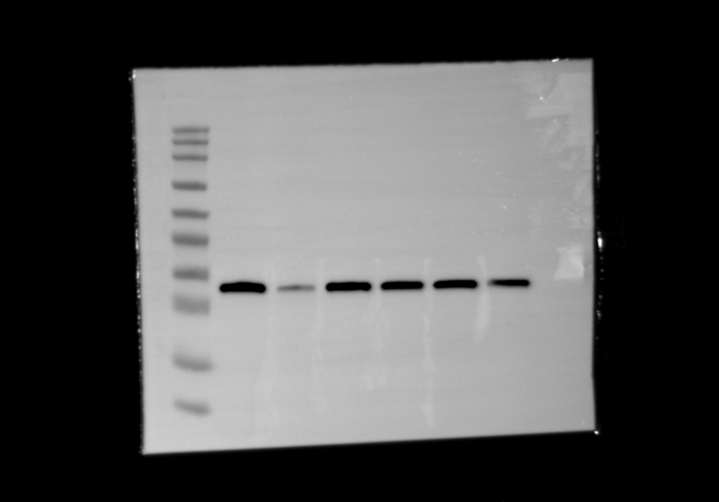


5.Interest protein-FTL


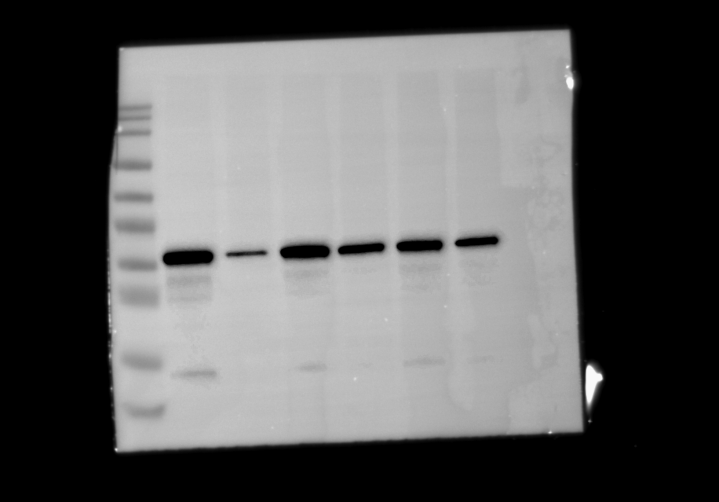


6.Interest protein-SLC7A11


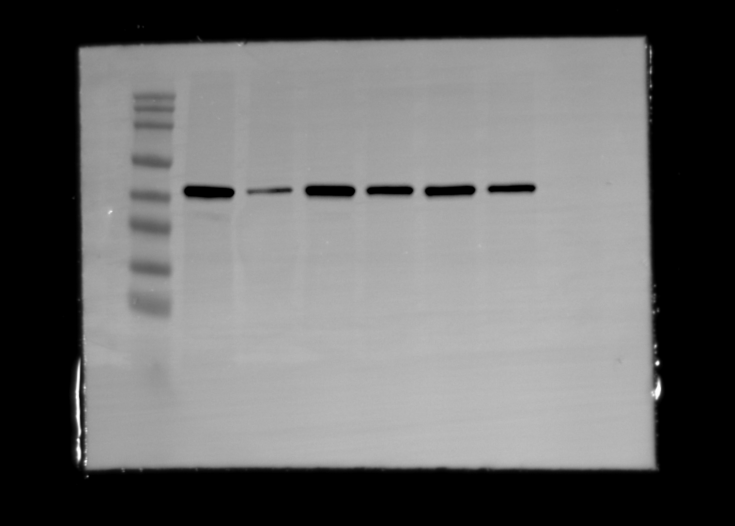


7.Interest protein-GPX4


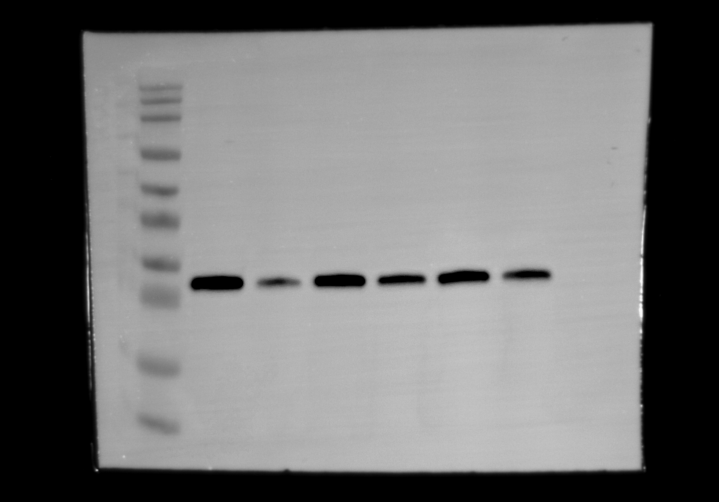


8.Interest protein-ACSL4


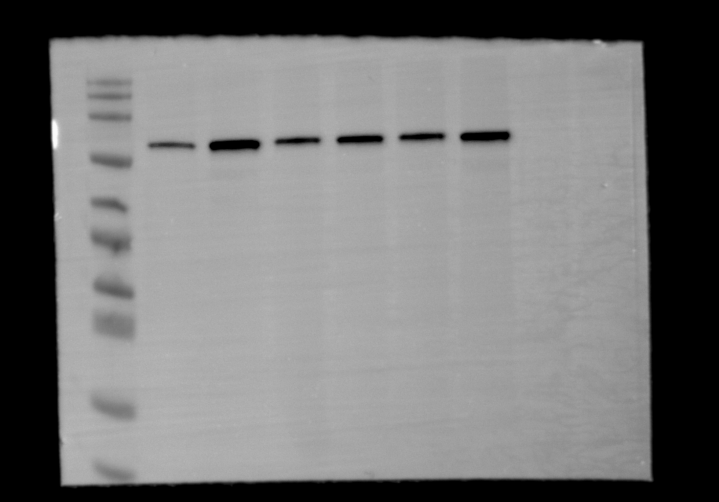


9.Interest protein-LOX


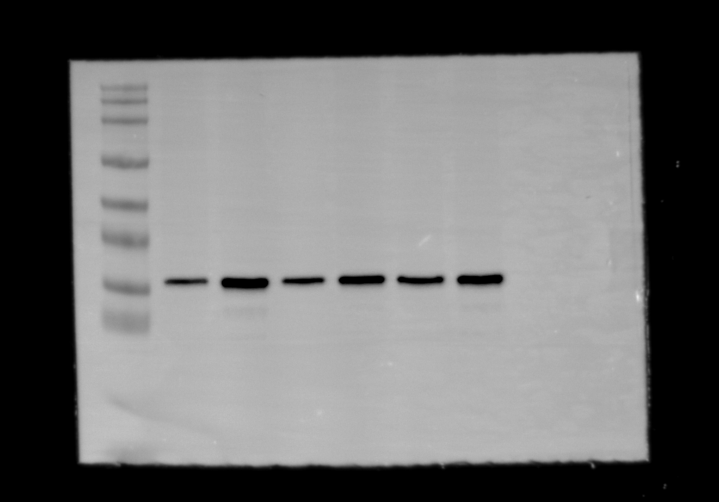


10.Interest protein-LPCAT3


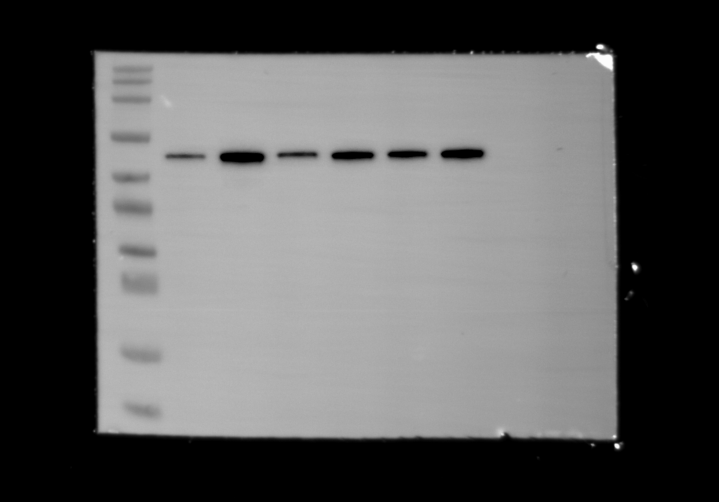

Supplement: Supplementary file 1 [file Data_Sheet_1.docx]
