# Supplementary material for: Mechanistic study on electroacupuncture-regulated circadian autophagy for inhibiting ferroptosis in hippocampal neurons and alleviating depression-like behaviors in adulthood induced by early chronic sleep deprivation
Source: Front Neurol. 2025 Nov 7;16:1680606. doi: 10.3389/fneur.2025.1680606 (PMC12621143; doi:10.3389/fneur.2025.1680606)

Supplementary Material

# Supplementary Figures and Tables

## Supplementary Figures

**Supplementary Figure 1.** Experimental procedure diagram**
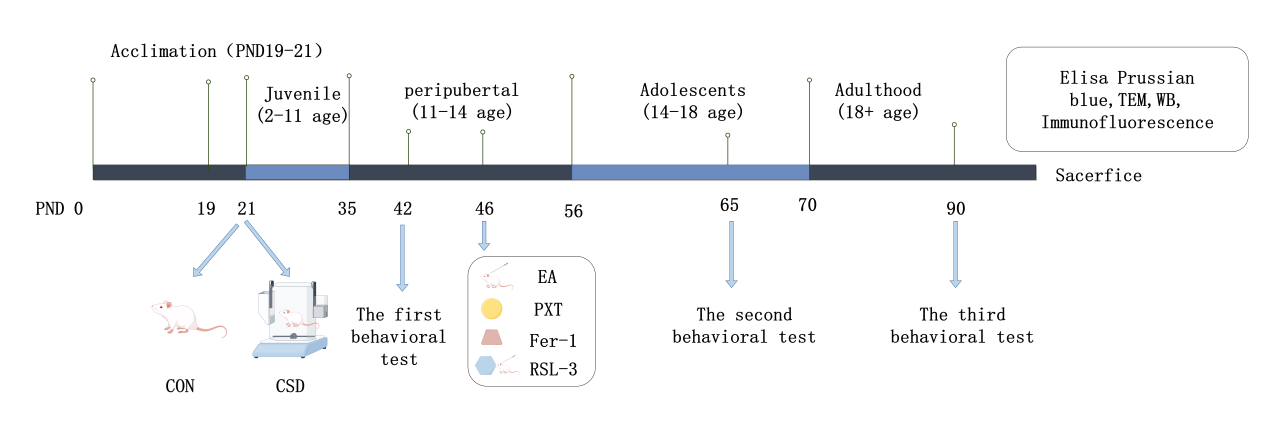
**

(ID:ROSWA19a14 www.home-for-researchers.com)


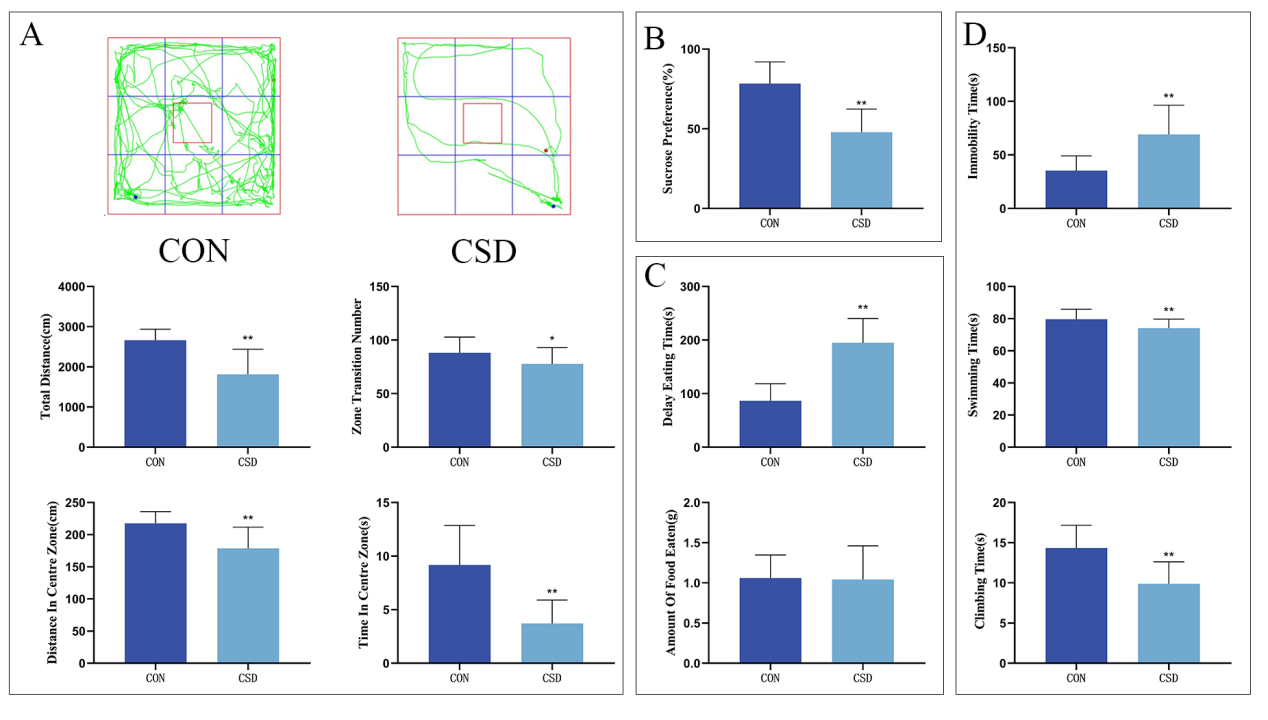


**Supplementary Figure 2.** (A) From top to bottom: OFT trajectory maps of rats in each group on PND 43, total distance traveled by rats in the open field, total number of grid crossings by rats in the open field, total distance traveled by rats in the central area, and time spent by rats in the central area. (B) Sucrose preference rates of rats in each group on PND 42. (C) Latency to feed and amount of food consumed by rats on PND 44. (D) Immobility ratio, swimming duration ratio, and struggling duration ratio of rats on PND 45. (CON group, n=12; CSD group, n=60). ^*^*P* < 0.05, ^**^*P* < 0.01 compared with the CON group; ^#^*P* < 0.05, ^##^*P* < 0.01 compared with the CSD group.


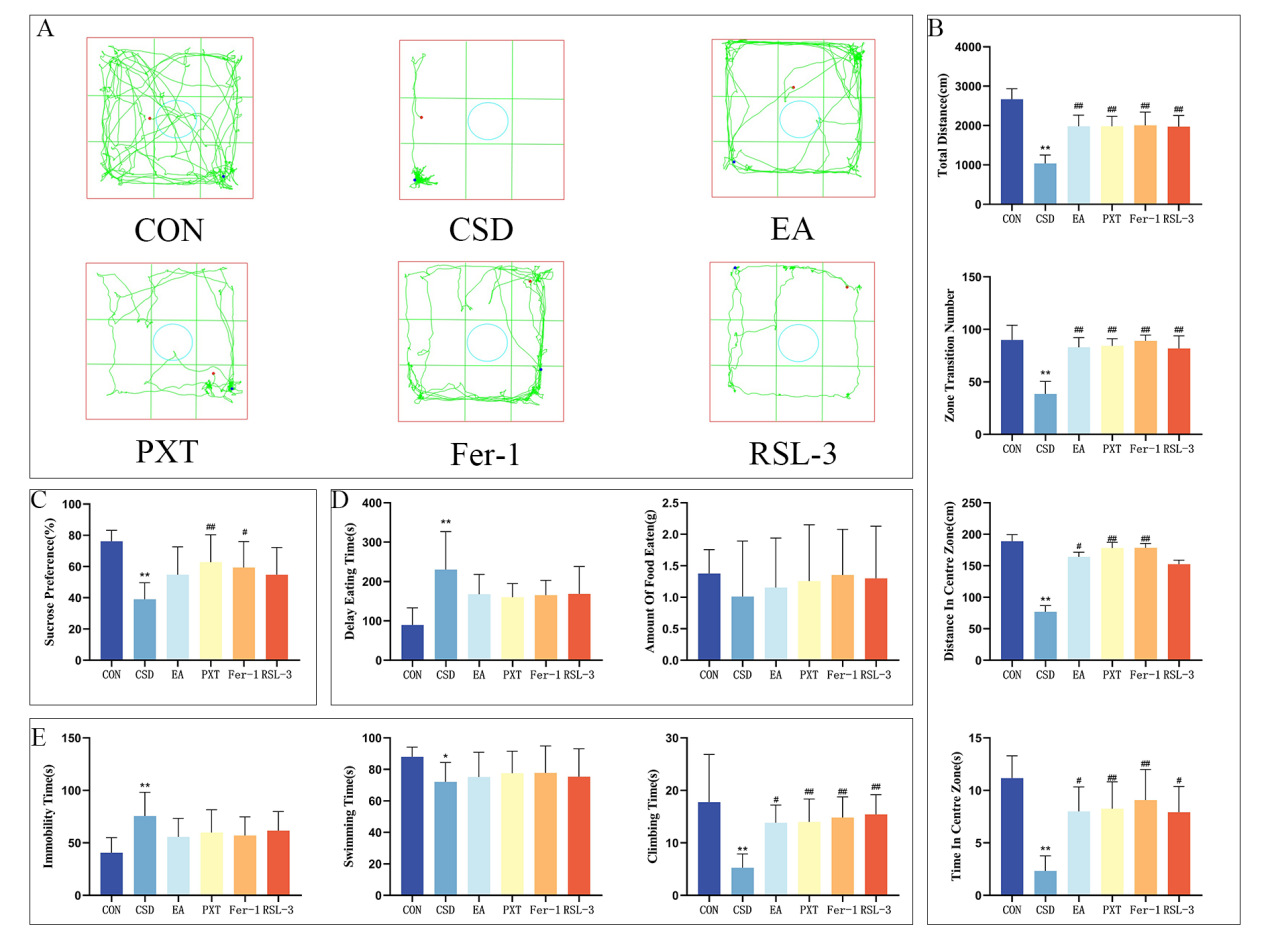


**Supplementary Figure 3.** (A) OFT trajectory maps of rats in each group on PND 66. (B) Total distance traveled by rats in the open field, total number of grid crossings by rats in the open field, total distance traveled by rats in the central area, and time spent by rats in the central area on PND 66. (C) Sucrose preference rates of rats in each group on PND 65. (D) Latency to feed and amount of food consumed by rats on PND 67. (E) Immobility ratio, swimming duration ratio, and struggling duration ratio of rats on PND 68. n = 12 per group. ^*^*P* < 0.05, ^**^*P* < 0.01 compared with the CON group; ^#^*P* < 0.05, ^##^*P* < 0.01 compared with the CSD group.


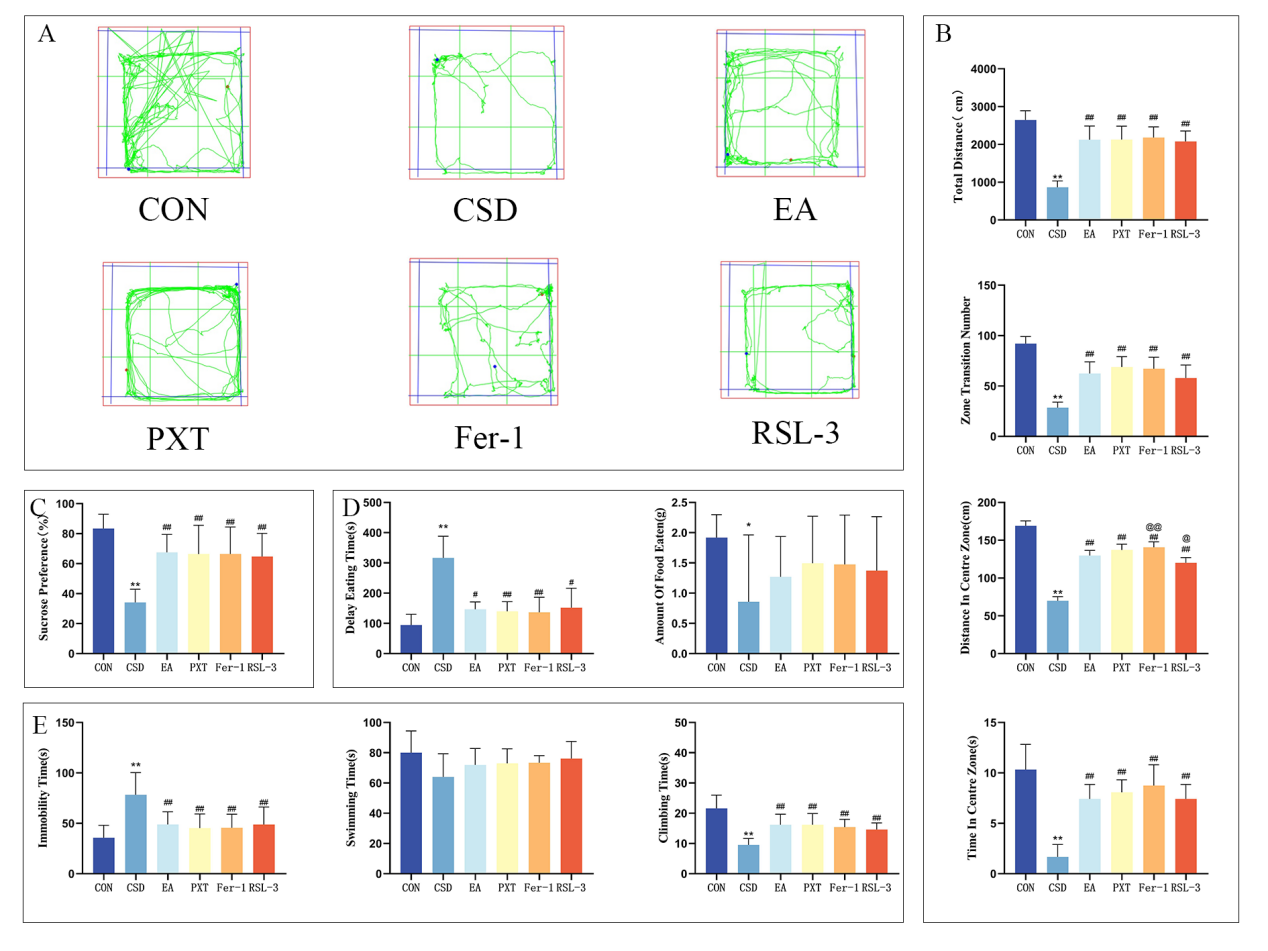


**Supplementary Figure 4.** (A) OFT trajectory maps of rats in each group on PND 91. (B) Total distance traveled by rats in the open field, total number of grid crossings by rats in the open field, total distance traveled by rats in the central area, and time spent by rats in the central area on PND 91. (C) Sucrose preference rates of rats in each group on PND 90. (D) Latency to feed and amount of food consumed by rats on PND 92. (E) Immobility ratio, swimming duration ratio, and struggling duration ratio of rats on PND 93. n = 12 per group.^*^*P* < 0.05, ^**^*P* < 0.01 compared with the CON group; ^#^*P* < 0.05, ^##^*P* < 0.01 compared with the CSD group; ^@^*P* < 0.05, ^@@^*P* < 0.01 compared with the EA group.


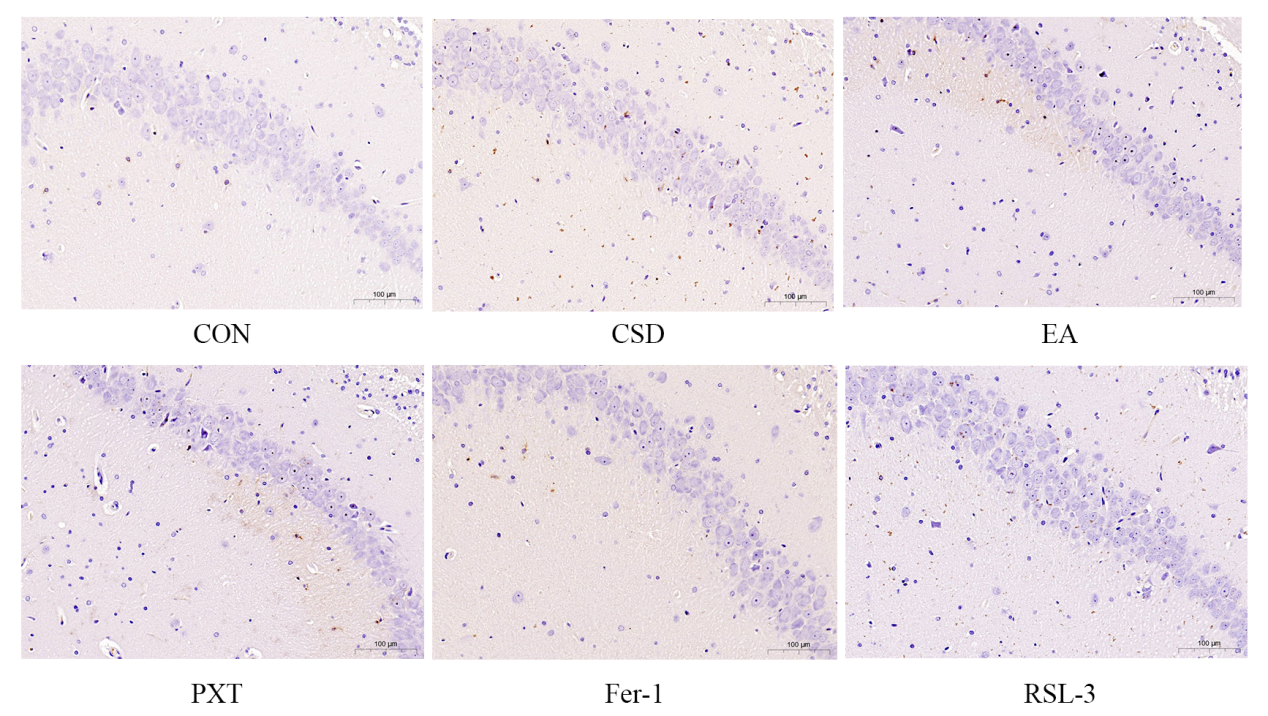


**Supplementary Figure 5.**The iron deposition in the hippocampus of rats in each group is shown in the figure.(scale bar: 100 μm, n = 3).


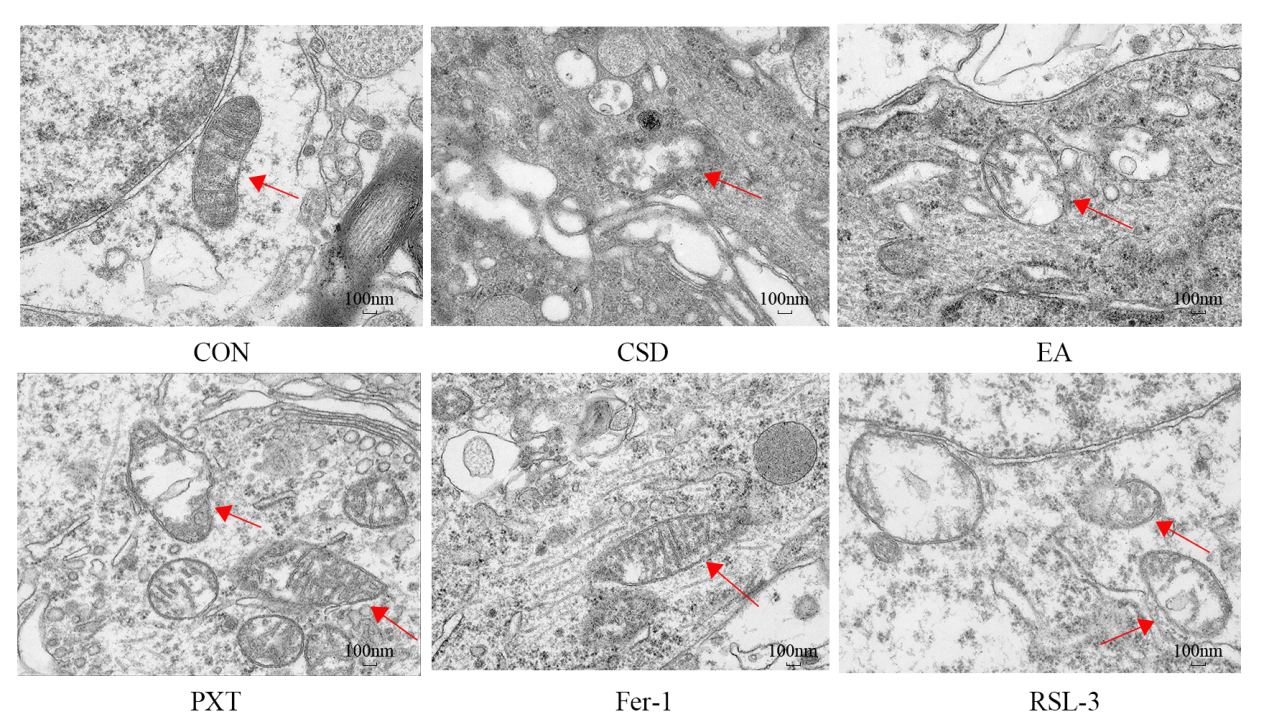


**Supplementary Figure 6.** TEM imaging of the mitochondrial ultrastructure and autophagy in hippocampal neurons in rats (scale bar: 100 nm, n = 3).


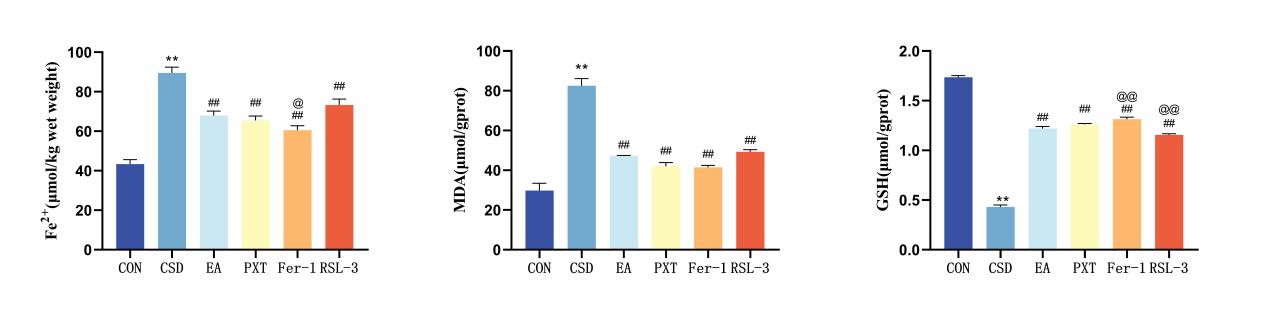


**Supplementary Figure 7.** Levels of Fe^2+^, GSH, and MDA in the hippocampal tissue of rats in each group (n = 3). ^*^*P* < 0.05, ^**^*P* < 0.01 vs. CON group; ^#^*P* < 0.05, ^##^*P* < 0.01 vs. CSD group; ^@^*P* < 0.05, ^@@^*P* < 0.01 vs. EA group.


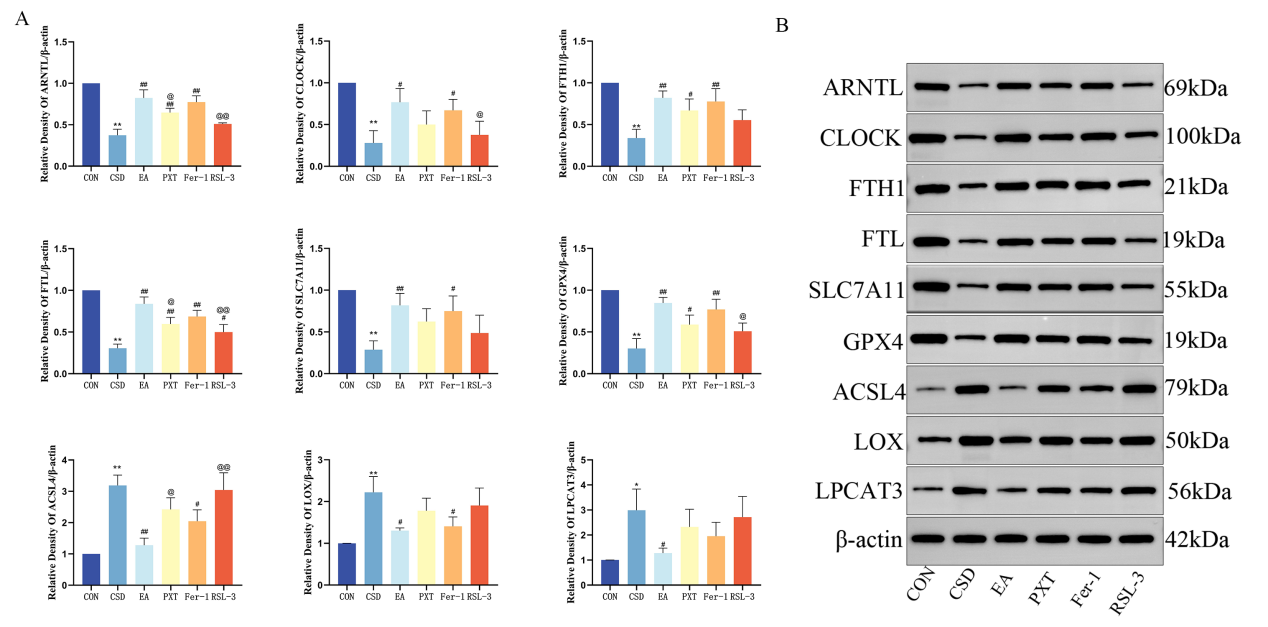


**Supplementary Figure 8.** (A) Relative expression levels of target proteins. (B) Representative Western blot bands (n = 3). ^*^*P* < 0.05, ^**^*P* < 0.01 vs. CON group; ^#^*P* < 0.05, ^##^*P* < 0.01 vs. CSD group; ^@^*P* < 0.05, ^@@^*P* < 0.01 vs. EA group.


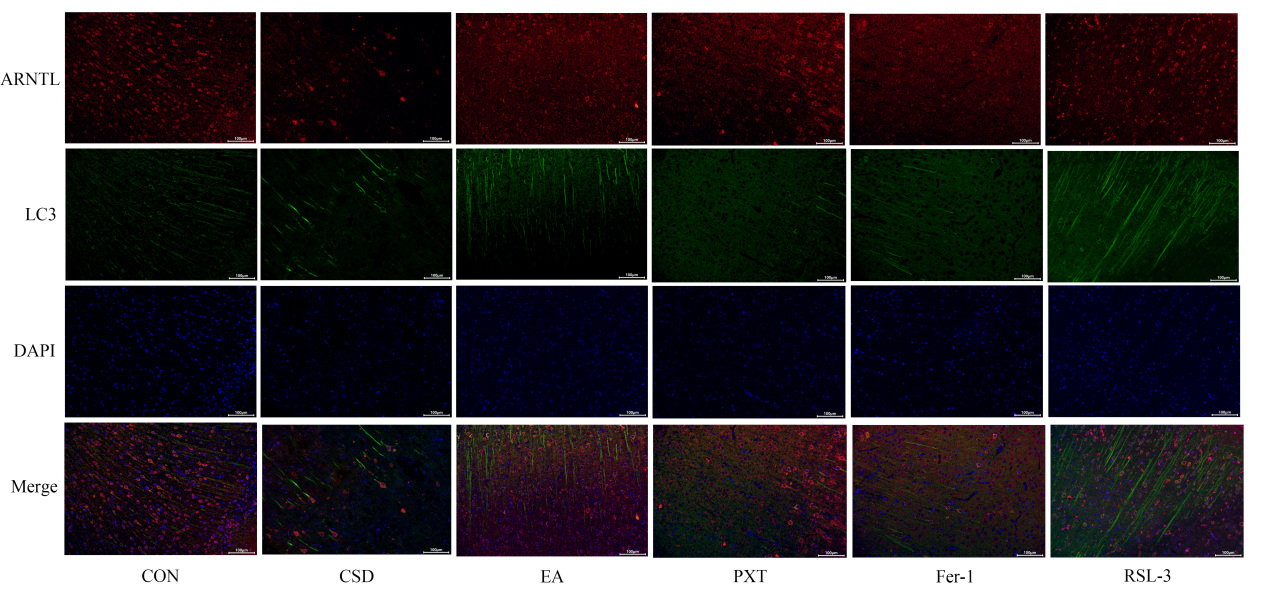


**Supplementary Figure 9.** Colocalization of circadian clock autophagy protein ARNTL with autophagosomes in rat hippocampal tissue of each group (n = 3).


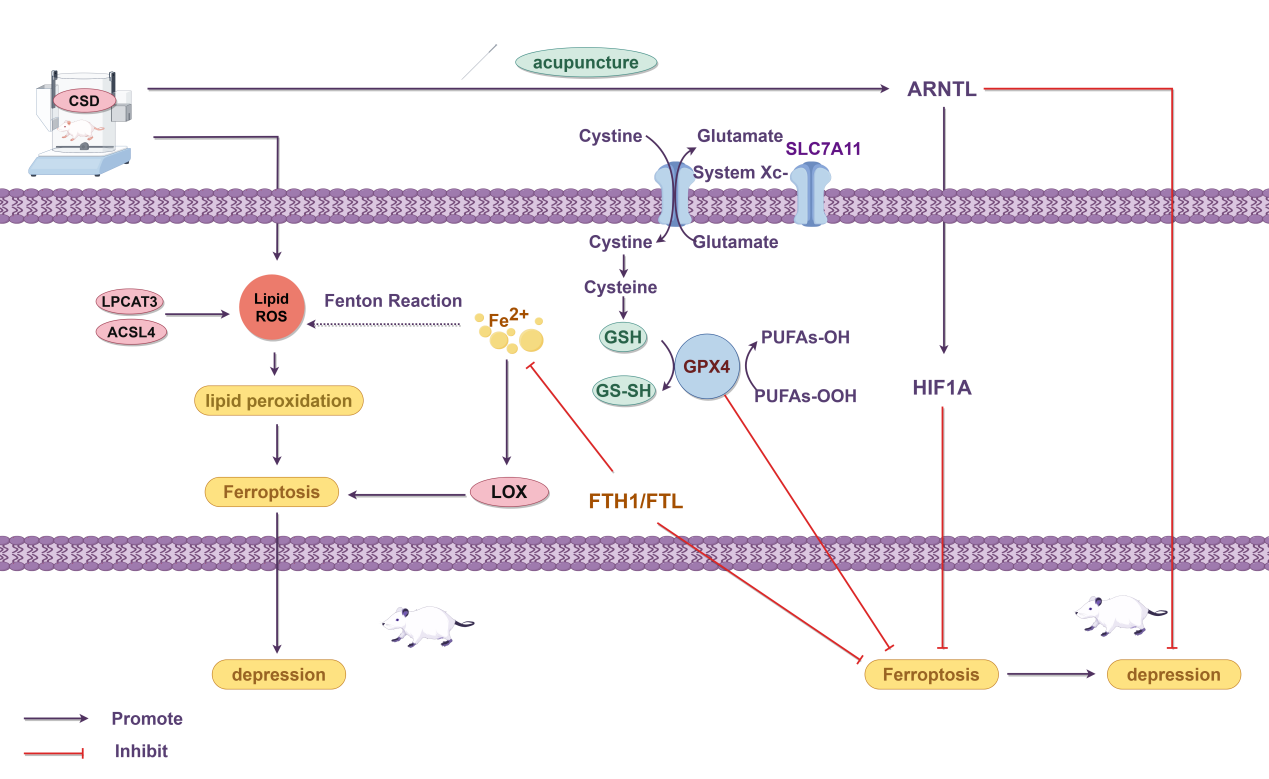


**Supplementary Figure 10.** Mechanism by which electroacupuncture regulates circadian autophagy to inhibit hippocampal neuronal ferroptosis and alleviate depression-like behaviors induced by early chronic sleep deprivation in adulthood.

(ID:YYSSW16176 www.home-for-researchers.com)

**Supplementary Table 1.** Levels of Fe^2+^, GSH, and MDA in hippocampal tissue of rats in each group (n = 3).

| Group | Fe^2+^ (μmol/kg wet weight) | MDA (μmol/g prot) | GSH (μmol/g prot) |
| --- | --- | --- | --- |
| CON | 43.36 ± 1.30 | 29.75 ± 2.15 | 1.74 ± 0.01 |
| CSD | 89.54 ± 1.70^**^ | 82.54 ± 2.08^**^ | 0.43 ± 0.01^**^ |
| EA | 67.92 ± 1.30^##^ | 47.21 ± 0.14^##^ | 1.22 ± 0.01^##^ |
| PXT | 65.47 ± 1.30^##^ | 41.97 ± 1.11^##^ | 1.26 ± 0.01^##^ |
| Fer-1 | 60.55 ± 1.30^##@^ | 41.43 ± 0.59^##^ | 1.31 ± 0.01^##@@^ |
| RSL-3 | 73.33 ± 1.70^##^ | 49.29 ± 0.61^##^ | 1.16 ± 0.01^##@@^ |

^*^*P* < 0.05, ^**^*P* < 0.01 vs. CON group; ^#^*P* < 0.05, ^##^*P* < 0.01 vs. CSD group; ^@^*P* < 0.05, ^@@^*P* < 0.01 vs. EA group.

**Supplement the original image of the WB.**

The first experiment

1.Internal reference protein-ACTIN


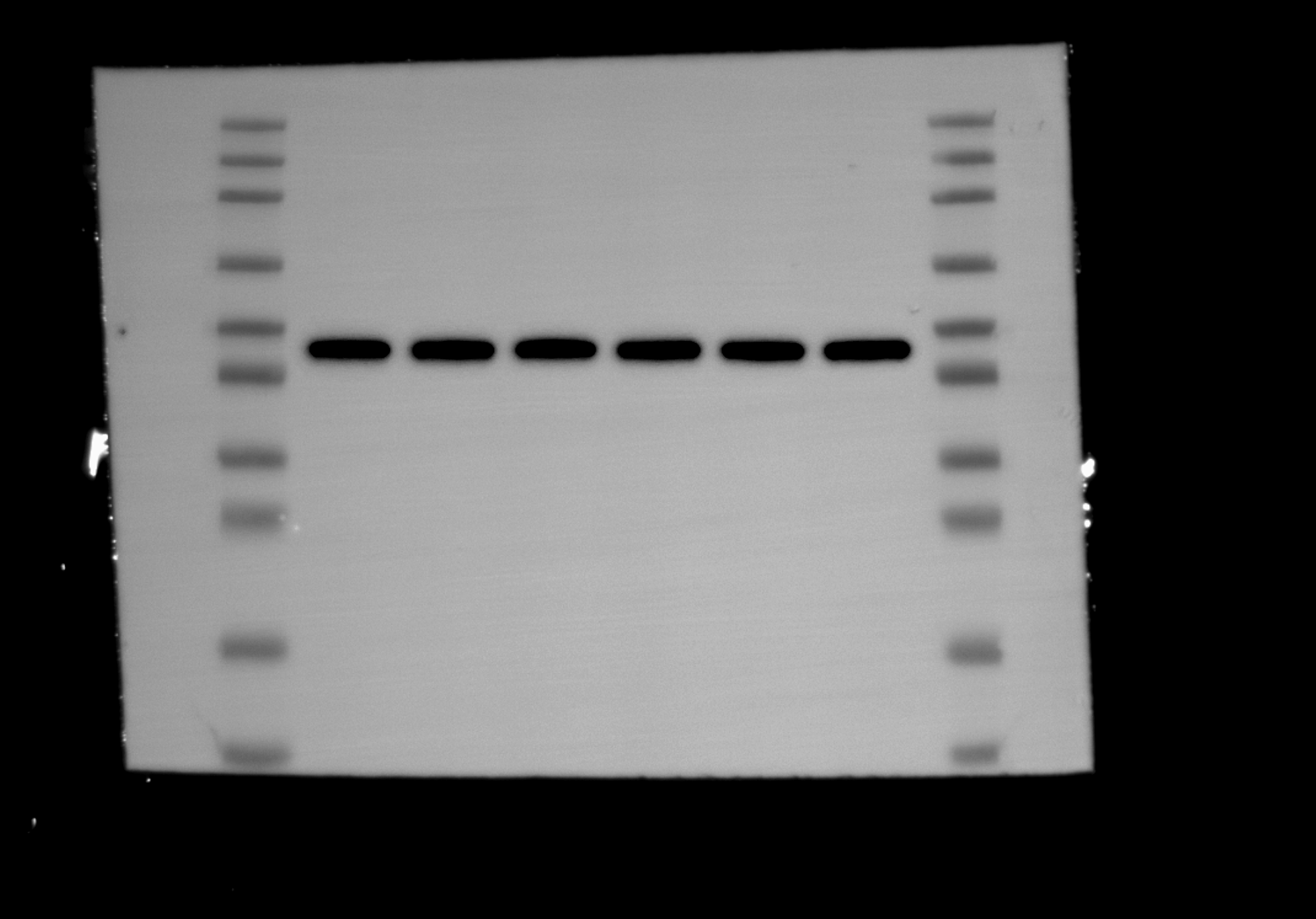


2.Interest protein-ARNTL


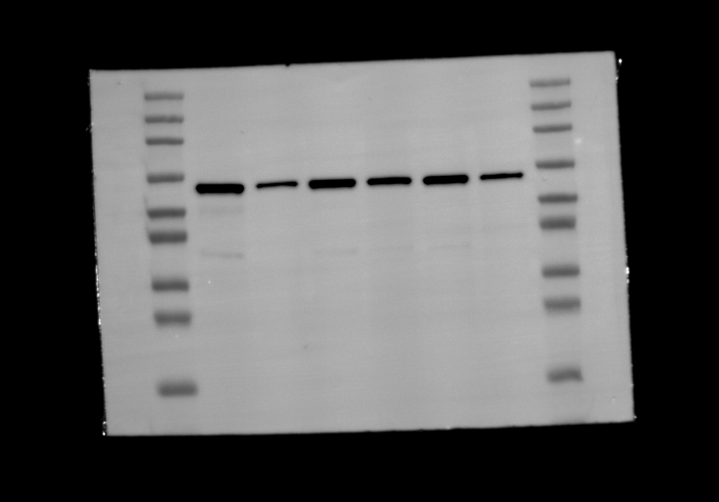


3.Interest protein-CLOCK


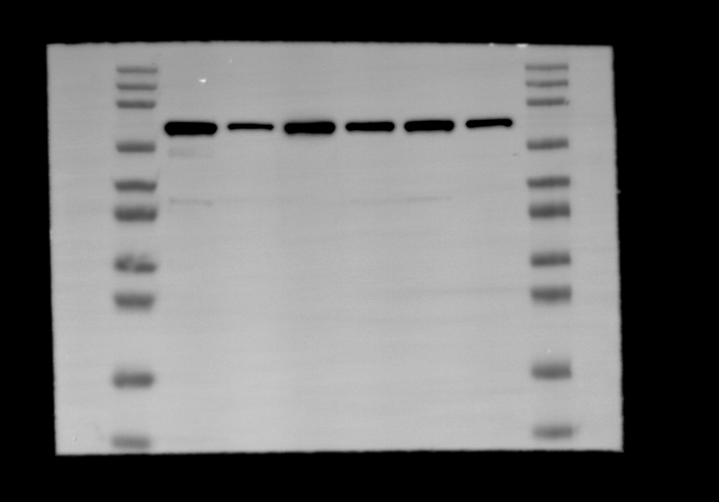


4.Interest protein-FTH1


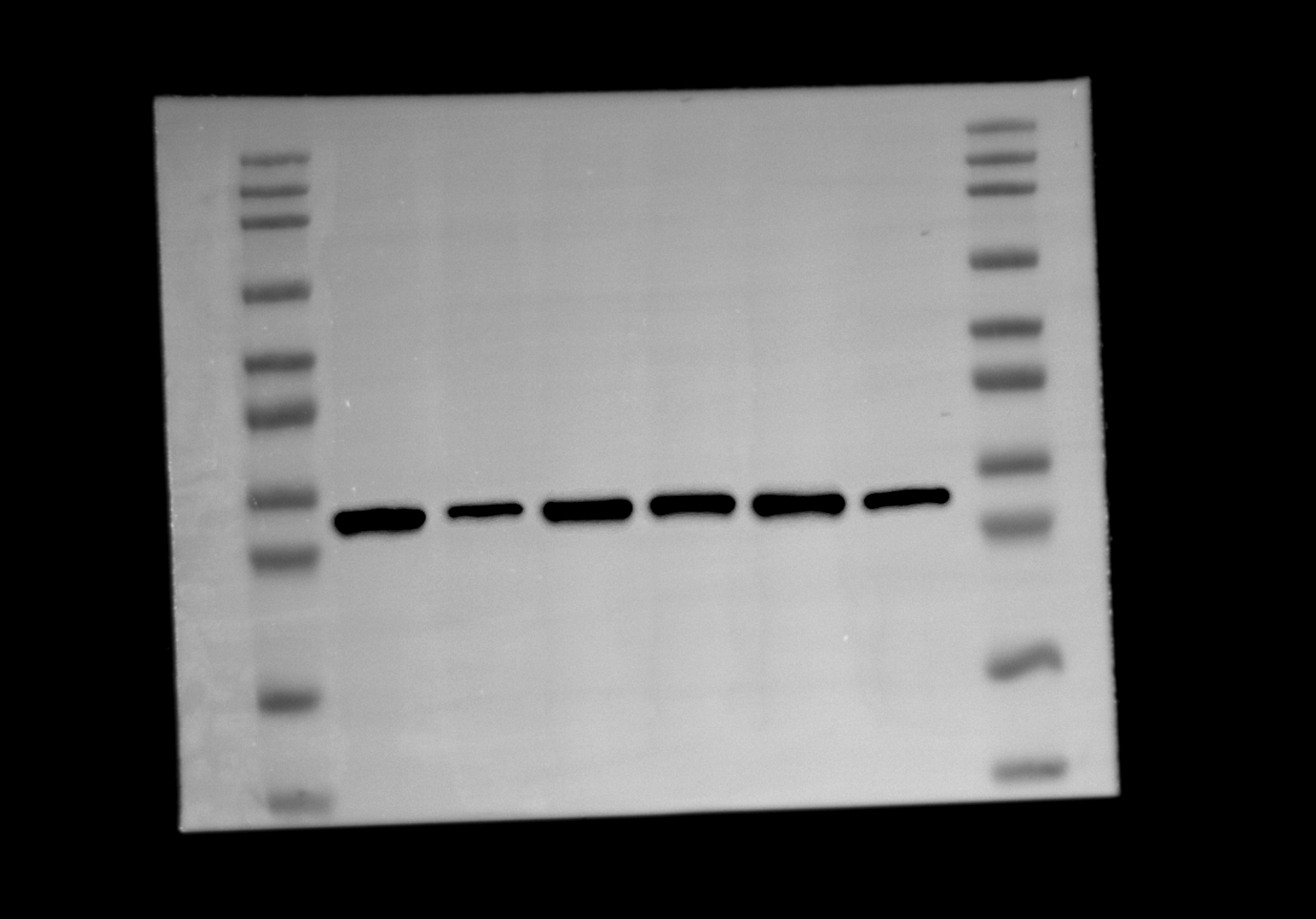


5.Interest protein-FTL


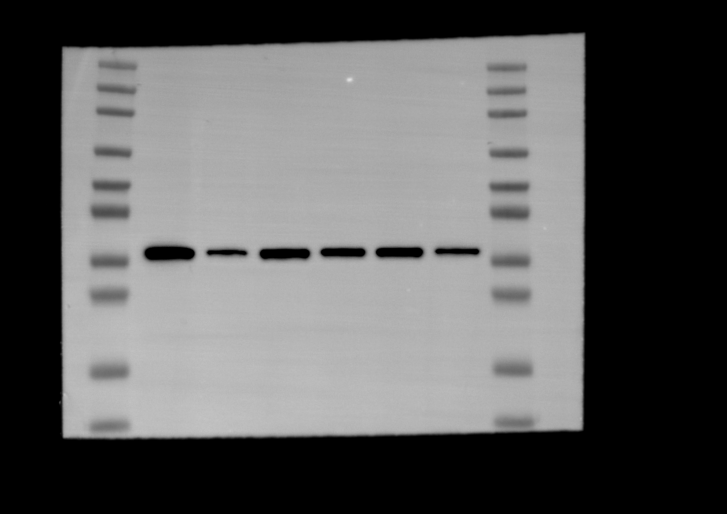


6.Interest protein-SLC7A11


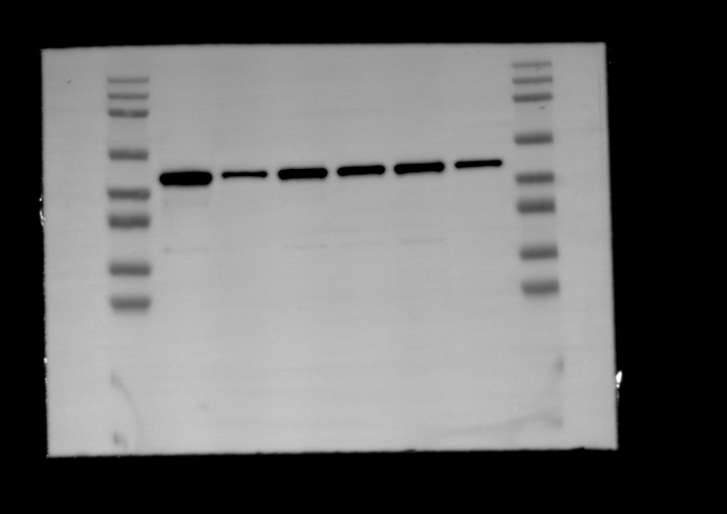


7.Interest protein-GPX4


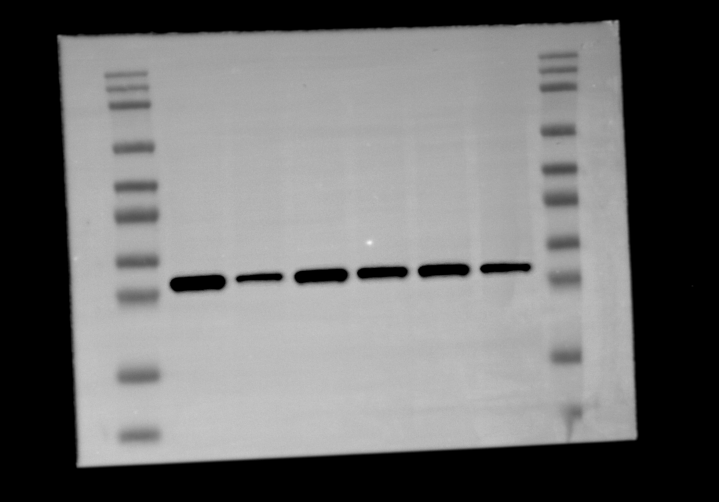


8.Interest protein-ACSL4


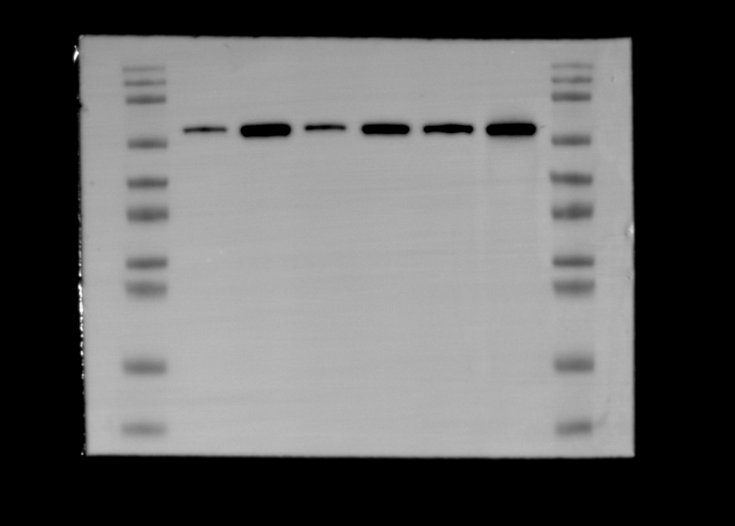


9.Interest protein-LOX


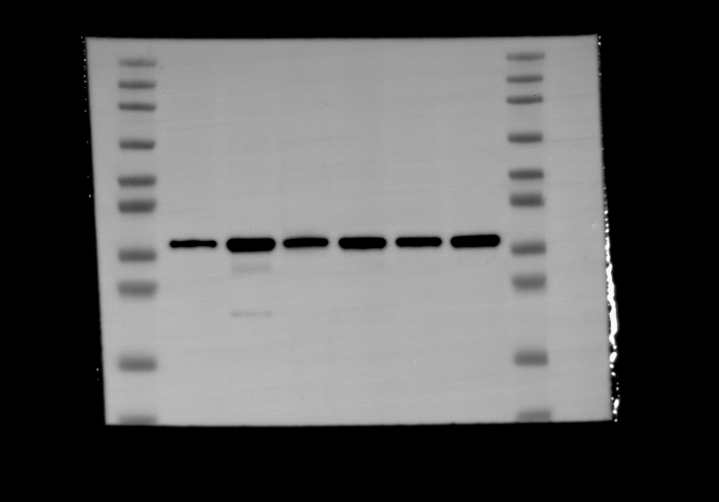


10.Interest protein-LPCAT3


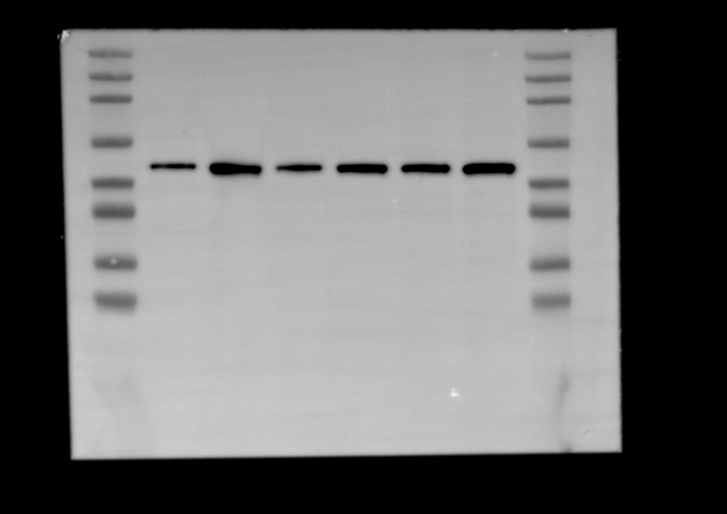


The second experiment

1.Internal reference protein-ACTIN


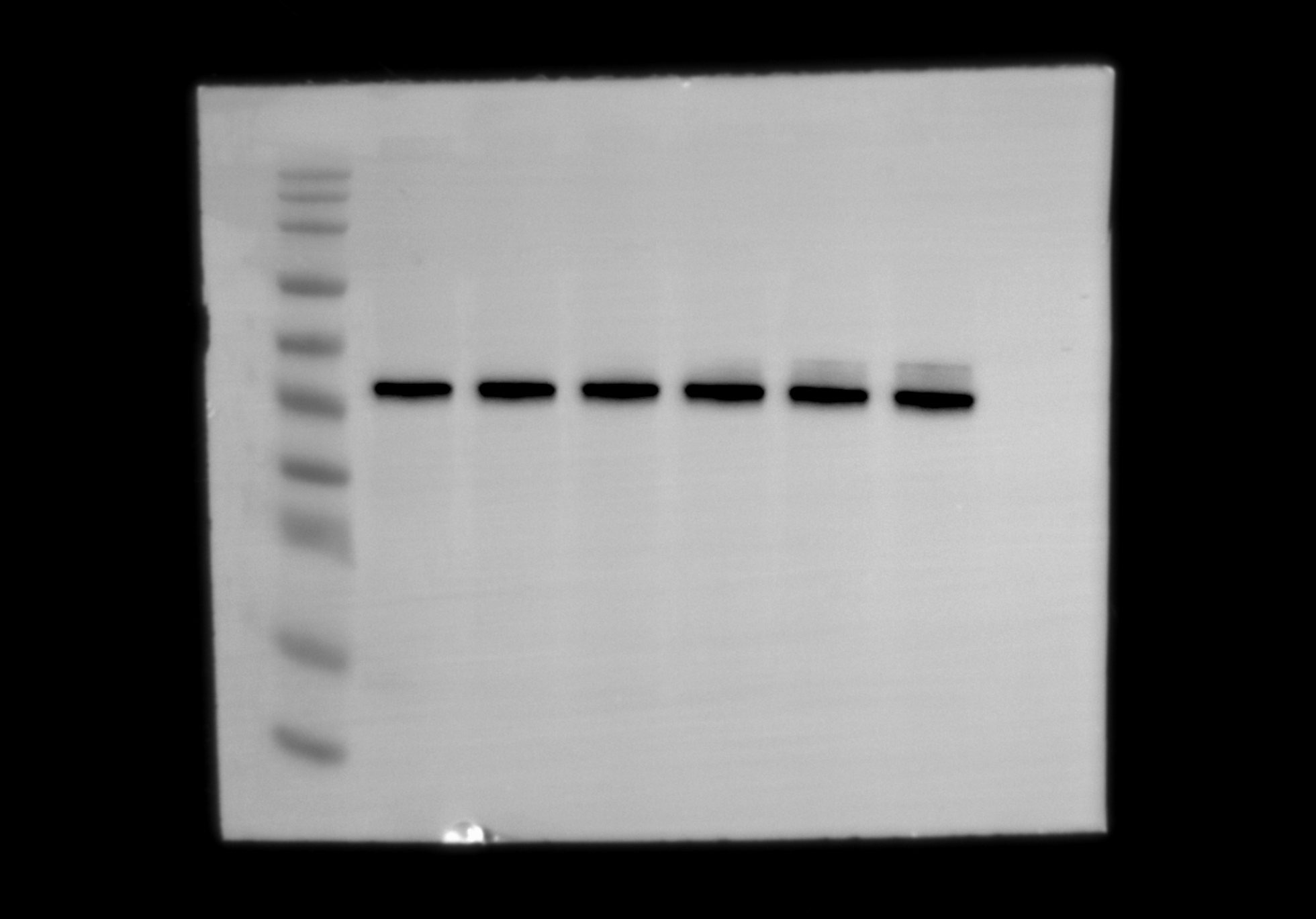


2.Interest protein-ARNTL


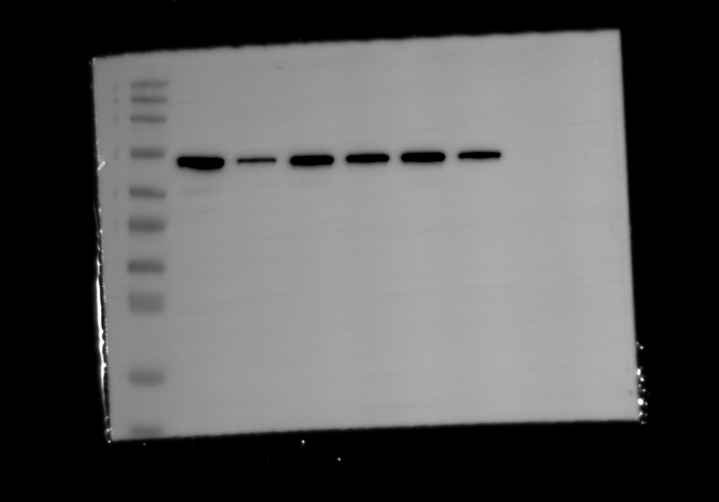


3.Interest protein-CLOCK


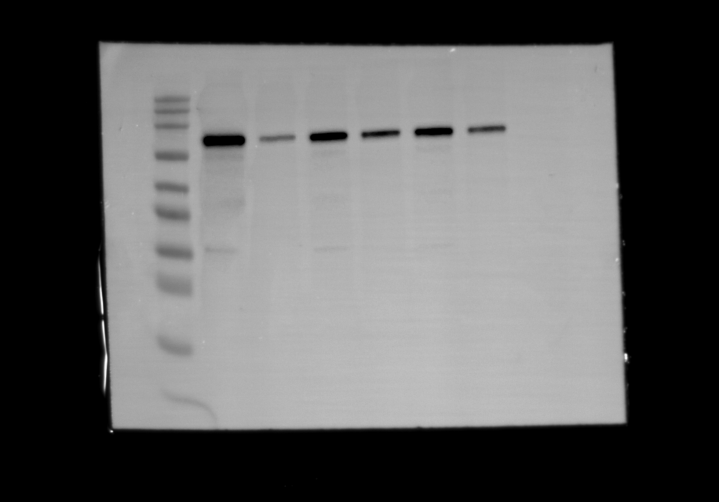


4.Interest protein-FTH1


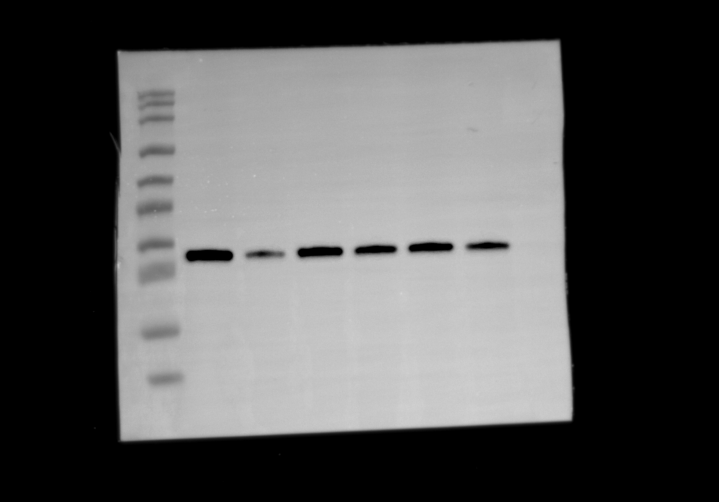


5.Interest protein-FTL


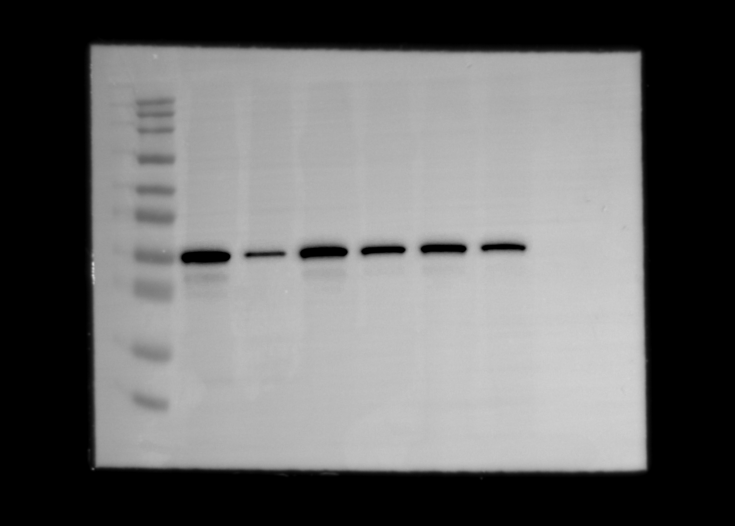


6.Interest protein-SLC7A11


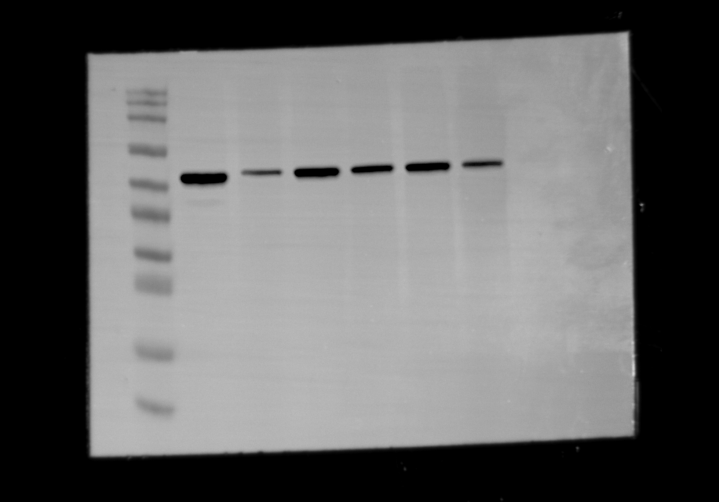


7.Interest protein-GPX4


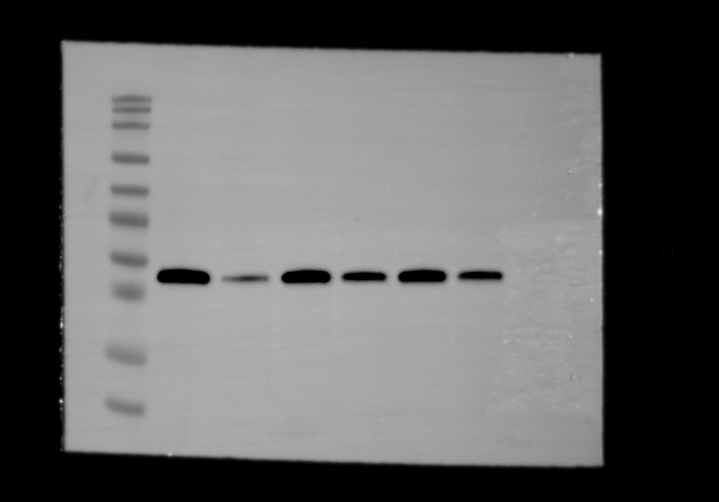


8.Interest protein-ACSL4


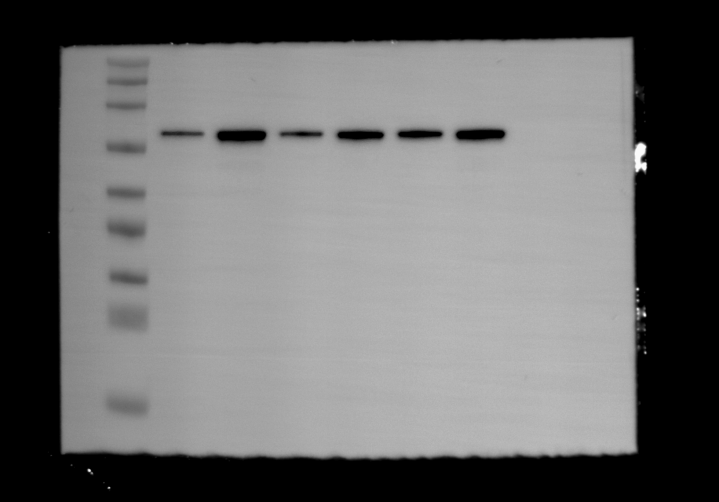


9.Interest protein-LOX


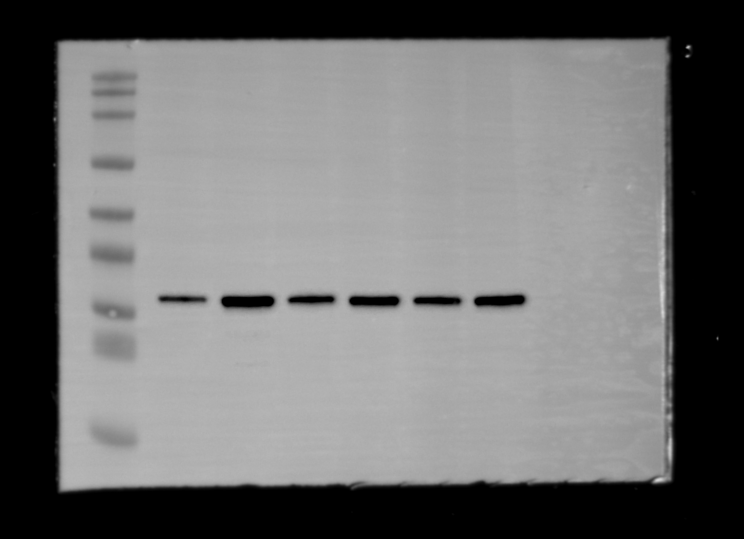


10.Interest protein-LPCAT3


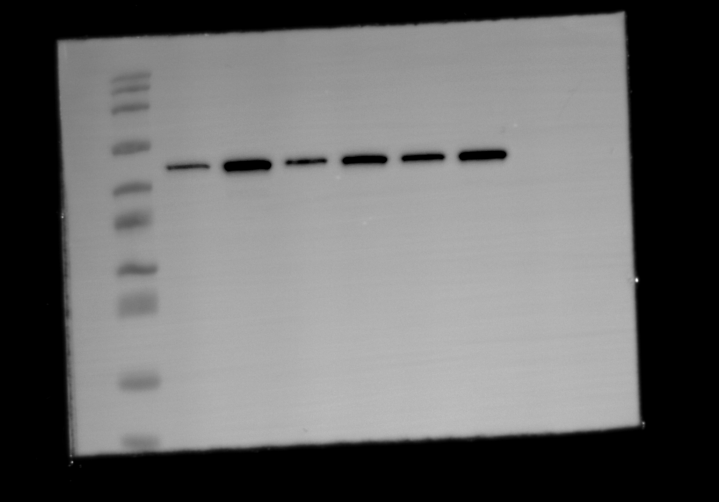


The third experiment

1.Internal reference protein-ACTIN


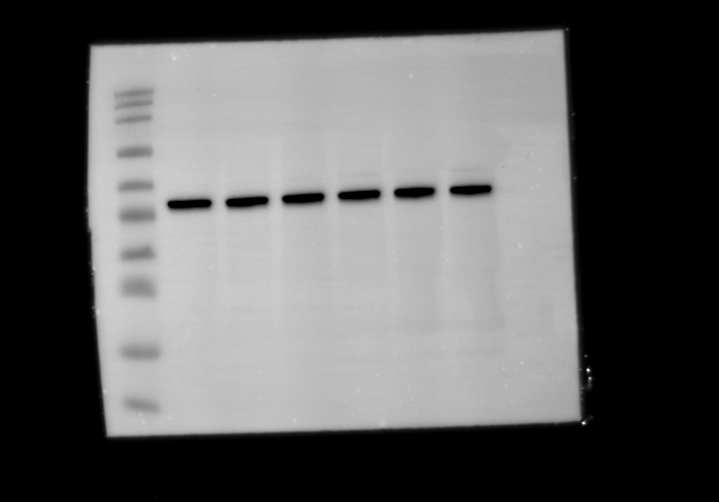


2.Interest protein-ARNTL


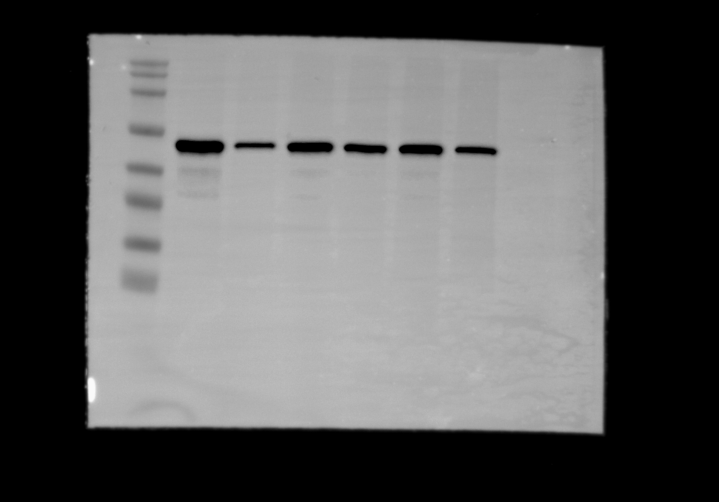


3.Interest protein-CLOCK


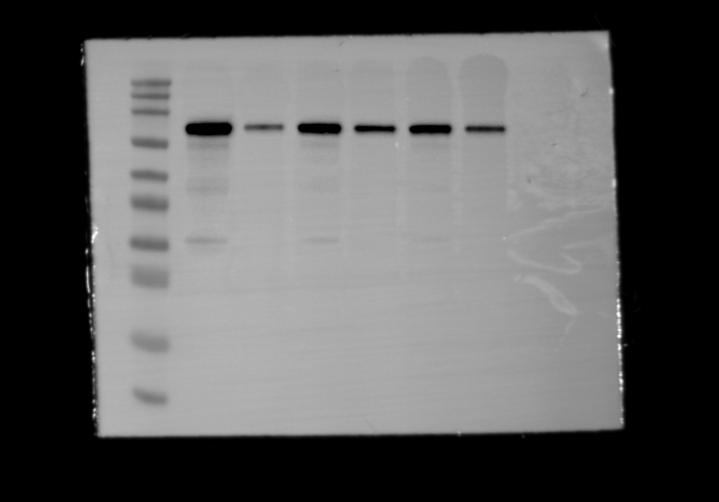


4.Interest protein-FTH1


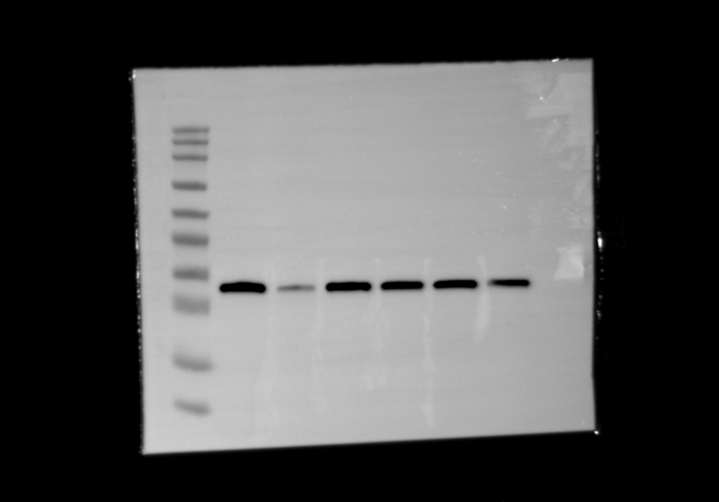


5.Interest protein-FTL


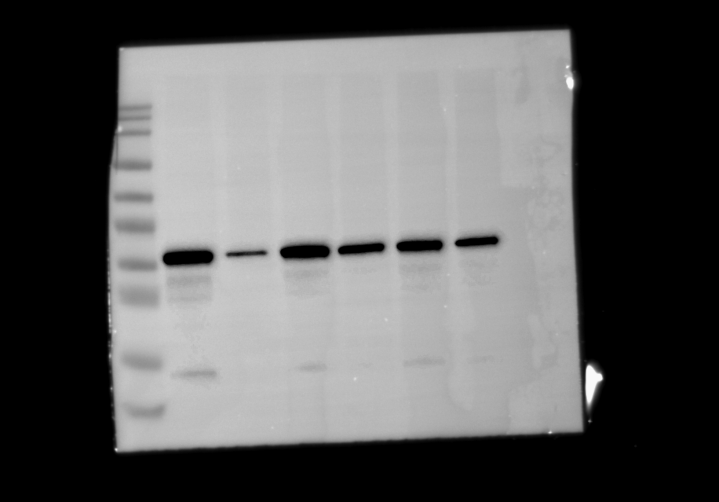


6.Interest protein-SLC7A11


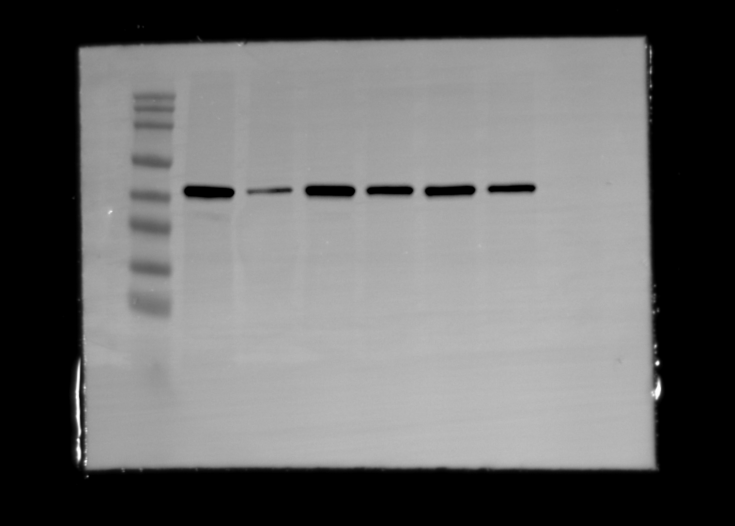


7.Interest protein-GPX4


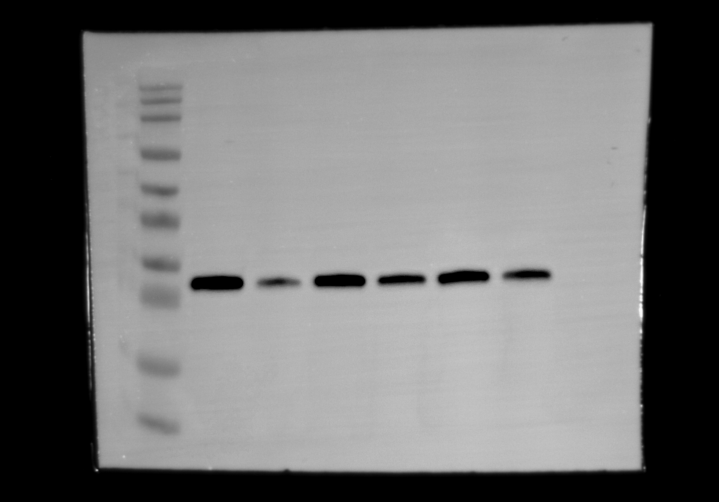


8.Interest protein-ACSL4


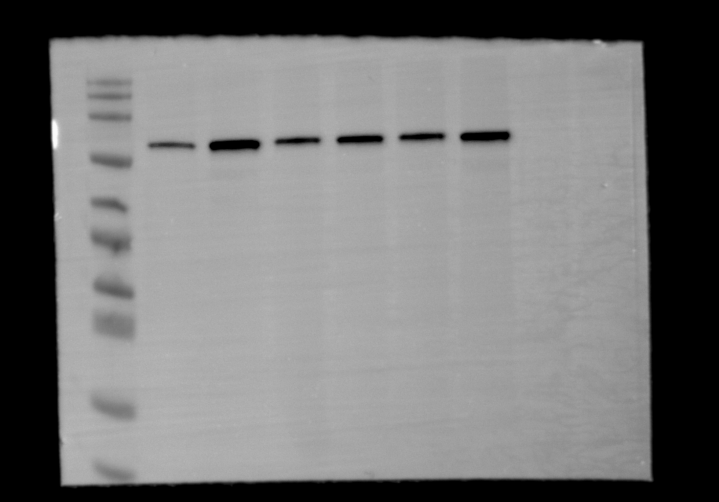


9.Interest protein-LOX


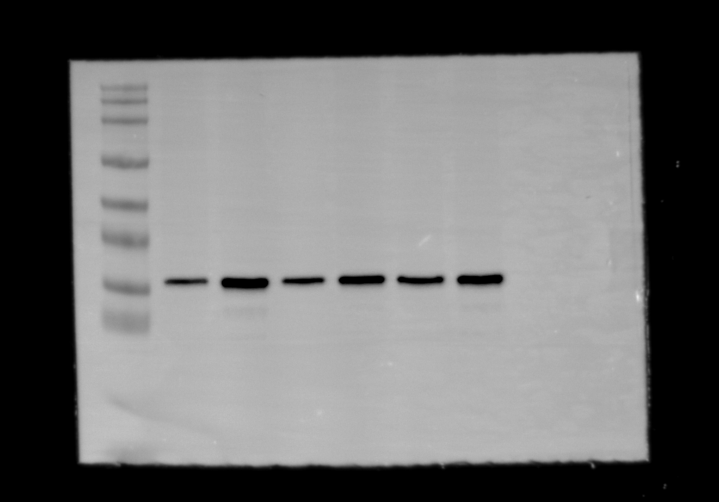


10.Interest protein-LPCAT3


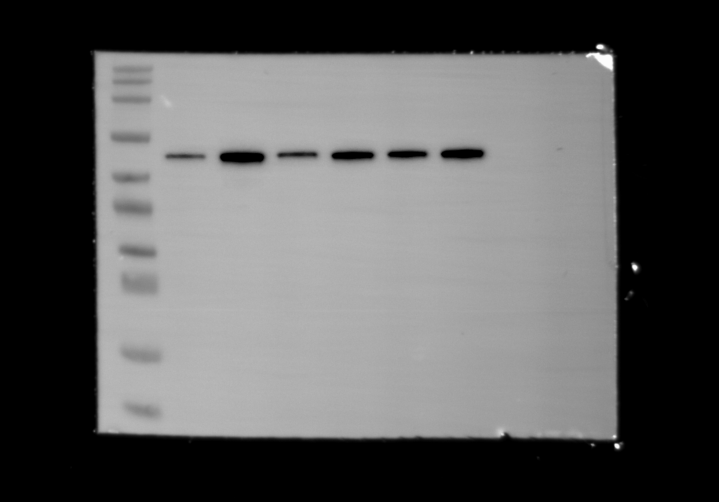

Supplement: Supplementary file 2 [file Table_1.docx]
